# Comparative Genomic Analysis of the Human Gut Microbiome Reveals a Broad Distribution of Metabolic Pathways for the Degradation of Host-Synthetized Mucin Glycans and Utilization of Mucin-Derived Monosaccharides

**DOI:** 10.3389/fgene.2017.00111

**Published:** 2017-08-29

**Authors:** Dmitry A. Ravcheev, Ines Thiele

**Affiliations:** Luxembourg Centre for Systems Biomedicine, University of Luxembourg Esch-sur-Alzette, Luxembourg

**Keywords:** human gut microbiome, comparative genomics, mucin glycans, carbohydrate utilization, metabolism reconstruction

## Abstract

The colonic mucus layer is a dynamic and complex structure formed by secreted and transmembrane mucins, which are high-molecular-weight and heavily glycosylated proteins. Colonic mucus consists of a loose outer layer and a dense epithelium-attached layer. The outer layer is inhabited by various representatives of the human gut microbiota (HGM). Glycans of the colonic mucus can be used by the HGM as a source of carbon and energy when dietary fibers are not sufficiently available. Both commensals and pathogens can utilize mucin glycans. Commensals are mostly involved in the cleavage of glycans, while pathogens mostly utilize monosaccharides released by commensals. This HGM-derived degradation of the mucus layer increases pathogen susceptibility and causes many other health disorders. Here, we analyzed 397 individual HGM genomes to identify pathways for the cleavage of host-synthetized mucin glycans to monosaccharides as well as for the catabolism of the derived monosaccharides. Our key results are as follows: (i) Genes for the cleavage of mucin glycans were found in 86% of the analyzed genomes, which significantly higher than a previous estimation. (ii) Genes for the catabolism of derived monosaccharides were found in 89% of the analyzed genomes. (iii) Comparative genomic analysis identified four alternative forms of the monosaccharide-catabolizing enzymes and four alternative forms of monosaccharide transporters. (iv) Eighty-five percent of the analyzed genomes may be involved in potential feeding pathways for the monosaccharides derived from cleaved mucin glycans. (v) The analyzed genomes demonstrated different abilities to degrade known mucin glycans. Generally, the ability to degrade at least one type of mucin glycan was predicted for 81% of the analyzed genomes. (vi) Eighty-two percent of the analyzed genomes can form mutualistic pairs that are able to degrade mucin glycans and are not degradable by any of the paired organisms alone. Taken together, these findings provide further insight into the inter-microbial communications of the HGM as well as into host-HGM interactions.

## Introduction

The colonic mucus layer is a dynamic and complex structure that is mainly composed of the glycoprotein mucin-2 (MUC2) (Johansson et al., 2008). MUC2 is characterized by abundant glycosylation. Highly variable glycan structures are O-linked to serine or threonine residues that are concentrated in so-called PTS (proline, threonine, and serine) domains. The mass of the glycosylated MUC2 protein is approximately 2.5 MDa, and more than 80% of this mass comes from carbohydrates (Lang et al., 2007). Glycosylated MUC2 can form a gel-like structure due to its N- and C-terminal domains that form numerous cross-links between cysteine residues (Johansson et al., 2013). Due to these cross-links, MUC2 forms a mucus structure. The colonic mucus consists of two layers, a loose outer layer and a dense epithelium-attached layer (Johansson et al., 2011). The inner layer acts as a physical barrier preventing bacteria from accessing the epithelium, whereas the outer layer is densely populated by various commensal microbes (Johansson et al., 2011, 2015; Li et al., 2015).

Interactions between the human gut microbiota (HGM) and colonic mucus are not limited just to residing of the microbes in the outer layer mucus. Thus, once in the outer mucus layer, gut microbes not only avoid washout by the contents flowing through the colon but also are able to access mucin glycans that can be used as sources of carbon and energy. Usually HGM microbes switch to mucin glycans in a shortage of dietary fibers, because multiple intestinal microbes can switch between dietary and host glycans (Mahowald et al., 2009). Both commensal and pathogenic microbes are able to degrade mucin glycans. The commensal microbes are usually able to cleave glycans with secreted glycosyl hydrolases (GHs) and lyases and further catabolize the derived monosaccharides, whereas most intestinal pathogens use monosaccharides released by commensal-secreted enzymes (Martens et al., 2008; Koropatkin et al., 2012; Marcobal et al., 2013; Cameron and Sperandio, 2015; Pacheco and Sperandio, 2015). Being both an environmental niche and a food source, the mucus layer plays a key role in shaping the HGM composition (Koropatkin et al., 2012; Johansson et al., 2015). In turn, the HGM is able to modulate mucus chemical composition via the degradation of glycans and peptides and the local release of bioactive factors that can change the expression patterns of the mucin-producing host cells (Deplancke and Gaskins, 2001). The degradation of mucin glycans is highly dependent on the host diet. Thus, decreased levels of fibers in the diet force the HGM to degrade more mucin glycans, resulting in the thinning and depletion of the mucus layer, which subsequently enhances host susceptibility to pathogens (Marcobal et al., 2013; Tailford et al., 2015; Desai et al., 2016). Additionally, depletion of the colonic mucus is associated with such disorders as Crohn's disease, celiac disease, colonic ischemia, compound exocytosis, and ulcerative colitis (Png et al., 2010; Joossens et al., 2011; Parmar et al., 2012; Johansson et al., 2013; Arike and Hansson, 2016; Cockburn and Koropatkin, 2016).

Because of the significant mucus-microbiota interaction, multiple HGM organisms have been analyzed for their abilities to degrade mucin glycans. To date, more than 50 mucus-degrading bacterial strains, generally belonging to *Akkermansia muciniphila, Bacteroides* spp., *Barnesiella intestinihominis, Bifidobacterium* spp., *Eubacterium* spp., and *Ruminococcus* spp. (Derrien et al., 2004; Sonnenburg et al., 2005; Martens et al., 2008; Comstock, 2009; Png et al., 2010; Kiyohara et al., 2012; Pudlo et al., 2015; Tailford et al., 2015; Desai et al., 2016), have been identified.

In this study, we analyzed the degradation of mucin glycans by the HGM using comparative genomic analysis. The comparative genomic analysis of sugar utilization by microbes is a recent but actively developing research area. A comparative genomic approach, combining a phylogenomics and genome-context based techniques (Osterman and Overbeek, 2003; Rodionov, 2007), has been previously applied for the analysis of sugar utilization in various microbial taxa, including multiple HGM strains (Leyn et al., 2012; Ravcheev et al., 2013; Zhang et al., 2015; Khoroshkin et al., 2016). Here, we used this comparative approach not to a set of related organisms, but to microorganisms cohabiting a certain environment, being the human intestine. Unlike functional analysis of metagenomes, this analysis is based on a reconstruction of metabolic pathways in individual genomes of microbes found in the studied environmental community and further prediction of possible interactions between different microorganisms. This approach has not previously been used for the detailed analysis of sugar utilization but it has been repeatedly used to reconstruct other metabolic pathways, including respiration (Ravcheev and Thiele, 2014), biosynthesis of B-vitamins (Magnúsdóttir et al., 2015), and quinones (Ravcheev and Thiele, 2016), as well central carbon metabolism and biosynthesis of amino acids and nucleotides (Magnúsdóttir et al., 2017), in multiple HGM genomes.

Here, we used a comparative genomics approach to analyze the degradation of mucin glycans as well utilization of the derived monosaccharides as carbon and energy sources in HGM genomes. Additionally, we predicted potential feeding pathways for mucin glycan-derived monosaccharides, the specificity of various HGM strains for different types of mucin glycans, and mutualistic relationships for the cleavage of mucin glycans by different HGM organisms.

## Materials and methods

### Analyzed genomes

The analyzed genomes were selected using the following steps. (1) All genomes listed in the Human Microbiome Project (HMP, http://www.hmpdacc.org/HMRGD/) as of 17.09.2015 and 459 genomes with the body site “Gastrointestinal tract” (i.e., isolated from the intestine) were selected. (2) All the genomes absent from the PubSEED (Overbeek et al., 2005; Disz et al., 2010) and Integrated Microbial Genomes (IMG) databases (Markowitz et al., 2014) were excluded. Among the remaining 397 genomes, 71 had a finished sequencing status whereas 326 others had a draft status (Table S1). These genomes represent 288 microbial species, 89 genera, 45 families, 19 orders, 14 classes, and 8 phyla. All the selected genomes, except 2 Archaea, are bacterial. The phyletic distribution of the analyzed genomes is in good agreement with that observed in various HGM (Eckburg et al., 2005; Goodman et al., 2011; Walker et al., 2011; Graf et al., 2015). Thus, the most represented phyla are Actinobacteria (37 genomes, 9.3% of the analyzed genomes), Bacteroidetes (69 genomes, 17.3%), Firmicutes (197 genomes, 49.6%), and Proteobacteria (71 genomes, 17.9%).

### Approach, tools, and databases

The PubSEED platform was used to annotate the genes responsible for the degradation of mucin glycans using the following comparative genomics approach. To avoid misannotation, all of the proteins with the same function were checked for orthology. Orthologs were defined as the best bidirectional hits that have a similar genomic context. To search for the best bidirectional hits, a BLAST algorithm (Altschul et al., 1997) implemented in PubSEED and the IMG platform was used with the following parameters, a score ≥150 bits, an *e*-value ≤ e^−50^, a protein identity and positives ≥25 and ≥40%, respectively, a query coverage at least 70%. To analyze genomic context, we used PubSEED and STRING v9.1 (Franceschini et al., 2013) along with phylogenetic trees for protein domains in MicrobesOnline (Dehal et al., 2010). To analyze protein domain structure, we searched the Pfam (Finn et al., 2014) and CDD (Marchler-Bauer et al., 2013) databases and the “Domains & Families” option of the MicrobesOnline platform with the threshold e^−30^. Additionally, functional annotations of the analyzed genes were performed using the UniProt (Magrane and Consortium, 2011), KEGG (Kanehisa et al., 2012), and MetaCyc (Caspi et al., 2014) databases.

After annotation of orthologs for the known analyzed genes, all the catabolic pathways (CPs) for monosaccharides utilization were checked for gaps. The CP was defined as sequence of reactions from intracellular monosaccharide to the intermediate of the central carbohydrate metabolism (glycolytic pathways or TCA cycle). A gap was defined as an absence of enzyme-encoding genes responsible for one or more reactions. Length of the gap was defined as a number of successive reactions corresponding to the absent genes. A CP was considered to be present in the organism if no more than two gaps were found and length of each gap was maximally one reaction. A search of the non-orthologous displacement was done for the gaps in the present pathways as well as for transporters absent in the presence of CP. If non-orthologous replacement was predicted, its orthologs were searched in all analyzed genomes. Afterwards, CPs were checked for gaps again while considering the identified non-orthologous replacements and the presence of CPs was re-evaluated.

To search for the protein homologs, a BLAST algorithm implemented in the PubSEED and the IMG platforms was used, with the following parameters, an *e*-value ≤ e^−20^, a protein identity ≥20%. Multiple protein alignments were performed using the MUSCLE v. 3.8.31 tool (Edgar, 2004a,b). Phylogenetic trees were constructed using the maximum-likelihood method with the default parameters implemented in PhyML-3.0 (Guindon et al., 2010). The obtained trees were visualized and midpoint-rooted using the interactive viewer Dendroscope, version 3.2.10, build 19 (Huson et al., 2007). To clarify the taxonomic affiliations of the analyzed genomes, the NCBI Taxonomy database (http://www.ncbi.nlm.nih.gov/taxonomy) was used. Analysis of gene occurrence was performed using the “Phylogenetic Profiler for Single Genes” option of the IMG platform.

The non-random distribution of the analyzed pathways, was tested using the Chi-squared test of observed frequencies of combinations with R (version 3.2.3).

All of the annotated genes are represented as a subsystem in PubSEED (http://pubseed.theseed.org/SubsysEditor.cgi; subsystem names are “Galactose utilization HGM,” “L-fucose utilization HGM,” “N-Acetylgalactosamine utilization HGM,” “N-Acetylglucosamine utilization HGM,” and “N-Acetylneuraminic acid utilization HGM”) and in Tables S2–S7. The protein sequences for the annotated genes in FASTA format are represented in the file Sequences S1 in the Supplementary Materials.

### Genome-context and phylogenomic approaches for functional annotation

The functions of some genes cannot be correctly annotated based on sequence similarity alone. Thus, a search of orthologs as described in Approach, Tools, and Databases is often incorrectly applied to large protein families containing proteins with different functions (Rodionov, 2007; Promponas et al., 2015). Additionally, the existence of non-orthologous displacements (Galperin and Koonin, 1998) require advanced approaches as genome context-based methods. Below, we describe non-trivial methods used in this work for functional annotation of the analyzed genes.

#### Annotation of the *fucK* and *fucA* genes

The main cause of misannotation for the *fuc* genes is their similarity to the genes for rhamnose utilization. Both the FucK and RhaB proteins belong to the FGGY family of carbohydrate kinases (Pfam: PF02782), whereas the FucA and RhaD proteins belong to the Aldolase_II family (Pfam: PF00596). We used the following steps to distinguish *fuc* and *rha* genes. (1) The *fucI* gene was used as a signature gene for the Fuc utilization pathway because this gene has no homologs among rhamnose-catabolizing enzymes. Orthologs for the FucI were found as described in Approach, Tools, and Databases. FucI proteins marked as reviewed in UniProt database were used as a query. Then, domain structures of all identified orthologs were analyzed and all the FucI orthologs demonstrated the presence of L-fucose isomerase domain (Pfam: PF07882) with *e*-value < e^−110^. The domain structure was used as an additional confirmation of orthology. (2) Phylogenetic maximum-likelihood trees were built for the FucK/RhaB- (Figure S1) and FucA/RhaD-like proteins (Figure S2) found in the analyzed genomes. (3) Genes that chromosomally clustered with *fucI* were annotated as *fucK* and *fucA*. (4) Single-copy genes found in genomes having *fucI* but lacking any *rha* genes were annotated as *fucK* and *fucA*. (5) The remaining non-annotated genes were annotated by their positions on the phylogenetic trees relative to the previously annotated genes.

#### Prediction of non-orthologs replacements for the Fcl fucose catabolism pathway

Non-orthologous displacements for genes for the Fcl pathways were found by an analysis of the chromosomal clusters in genomes having *fclBC* gene clusters but lacking the *fclA, fclD*, and *fclE* genes. The six genomes satisfying these criteria, were analyzed, *Bifidobacterium bifidum* NCIMB 41171, *Bifidobacterium breve* DSM 20213, *Bifidobacterium breve* HPH0326, *Bifidobacterium longum* ATCC 15697, *Bifidobacterium pseudocatenulatum* DSM 20438, and *Corynebacterium ammoniagenes* DSM 20306. Genes found to be chromosomally clustered with the *fclBC* were proposed to be non-orthologous replacements for the *fclADE* genes and their possible functions were predicted as described below.

The non-orthologs displacement for *fclE*, was predicted by similarity of the encoded protein to 4-hydroxy-tetrahydrodipicolinate synthase (DHDPS family, Pfam: PF00701). Because pyruvate is a product of FclE-catalyzed reaction (Yew et al., 2006) as well of reactions catalyzed by enzymes from DHDPS family (N-acetylneuraminate lyase and *trans*-o-hydroxybenzylidenepyruvate hydratase-aldolase), we concluded that the analyzed protein is an alternative form of FclE and named it FclE2.

The non-orthologous displacement for FclA and FclD proteins was predicted by analysis of protein families and phylogenetic trees. Thus, the *fclBC*-clustered gene was found to belong to the short-chain dehydrogenase family (Pfam: PF00106). Because this family includes experimentally analyzed FucA and FucD proteins from and *Burkholderia multivorans* (Hobbs et al., 2013), we constructed phylogenetic trees for FclA and FclD proteins as well short-chain dehydrogenase family proteins encoded in the *fclBC* chromosomal clusters (Figure S3). Because all the *fclBC*-clustered proteins formed a branch, separated from both FclD and FclA branches, we proposed that they have two functions, thus being non-orthologs replacements for both FclD and FclA. These proteins were were designated FclA2/FclD2.

#### Prediction of novel fucose-specific ABC transport system

Possible Fuc-specific ABC transport systems have been predicted by chromosomal clustering with the *fuc* genes in 25 analyzed genomes, including *Actinomyces* spp., *Clostridium* spp., *Coprococcus* spp., *Lachnospiraceae bacterium, Ruminococcus* spp., and *Enterobacter* spp. The closest experimentally analyzed homolog of the substrate-binding subunit of this system is the substrate-binding protein AraF of the arabinose-specific ABC system *from E. coli* (Johnson and Schleif, 2000). Additionally, genes of this system co-cluster together with the *fcl* genes in the genomes of *Streptomyces* sp. HGB0020, *Bifidobacterium longum* ATCC 15697, and *Bifidobacterium pseudocatenulatum* DSM 20438.

#### Annotation of homologous enzymes for GalNAc and GlcNAc metabolism

Previously it was found that at least some Firmicutes and Proteobacteria can utilize only galactosamine (GalN) but not GalNAc (Leyn et al., 2012; Zhang et al., 2015). The crucial feature of the GalNAc-utilizing microorganisms is the presence of the *agaA* gene for N-acetylgalactosamine-6-phosphate deacetylase. For proper pathway reconstruction, AgaA should be distinguished from the N-acetylglucosamine-6-phosphate deacetylase NagA, which is involved in GlcNAc utilization. For this purpose, we used the following pipeline. (1) The *agaS* and gene was selected as signature gene for the GalNAc utilization pathway, and *nagB* was used as a signature gene for the GlcNAc utilization pathway. These genes were selected because AgaS have no homologs in the GlcNAc utilization pathway and NagB has no homologs in GalNAc utilization pathway. (2) Orthologs for AgaS were found as described in Approach, Tools, and Databases. For a query, proteins marked as reviewed in UniProt database as well AgaS proteins annotated at the RegPrecise database (Novichkov et al., 2013) were used. The domain structures of all found AgaS orthologs were analyzed and all them demonstrated the presence of SIS_AgaS_like domain (NCBI CDD: cd05010) with *e*-value < e^−70^. Additionally, the phylogenetic maximum-likelihood tree for the AgaS proteins was constructed (Figure S4). The constructed tree was quite compact, did not include long branches, and its structure was in agreement with microbial taxonomy, which was used as an additional corroboration for orthology of all the predicted AgaS proteins. (3) Orthologs for the NagB were found as described in Approach, Tools, and Databases. For a query, proteins marked as reviewed in UniProt database as well AgaS proteins annotated at the RegPrecise database were used. All these orthologs belong to the GlcN6P_deaminase family (NCBI CDD: cd01399) with an *e*-value < e^−100^. (4) A phylogenetic maximum-likelihood tree was built for AgaA/NagA-like proteins (Figure S5) found in the analyzed genomes. (5) Genes chromosomally clustered with *agaS* were annotated as *agaA*, while genes chromosomally clustered with *nagB* were annotated as *nagA*. (6) Genes that co-occurred in the genomes with only *agaS* were annotated as *agaA*, and genes that co-occurred in the genomes with only *nagB* were annotated as *nagA*. (7) The remaining non-annotated genes were annotated by their positions on the phylogenetic trees relative to the previously annotated genes.

#### Annotation of the GalNAc-specific transporters

Three PTSs associated with various *aga* genes were previously identified, including a GalNAc-specific (AgaPTS), a GalN-specific (GamPTS), and a GnbPTS with multiple specificities (Leyn et al., 2012; Bidart et al., 2014; Zhang et al., 2015). The GnbPTS can transport and phosphorylate three different compounds: GalNAc, lacto-N-biose (Galβ-1,3-GlcNAc), and galacto-N-biose (Galβ-1,3-GalNAc).

Only AgaPTS and GnbPTS but not GamPTS are involved in GalNAc catabolism. We used the following steps to distinguish the various types of PTSs associated with *aga* genes. (1) Homologs were found for all the previously annotated EIIC components of the AgaPTS, GamPTS, and GnbPTS as described in Approach, Tools, and Databases. EIIC components were selected because these components are substrate-binding subunits. Query proteins were extracted from the RegPrecise database. (2) A phylogenetic maximal-likelihood tree was constructed for the EIIC components of the analyzed PTS systems (Figure S6). (3) The EIIC components clustered with the *gnbG* gene were annotated as binding GalNAc as well lacto- and galacto-N-biose. The *gnbG* gene encodes intracellular glycosyl-hydrolase, specific to lacto- and galacto-N-biose and clustered with the GnbPTS in all genomes previously analyzed (Bidart et al., 2014; Zhang et al., 2015). Additionally, the EIIC proteins clustered with the GnbG are clearly separated on the phylogenetic tree (Figure S6) and thus can be easily distinguished from their homologs. (4) The AgaS protein was used as a signature for the GalNAc and GalN utilization pathways, whereas the presence or absence of AgaA protein was used as a signature for GalNAc or GalN utilization, respectively (see Annotation of Homologous Enzymes for GalNAc and GlcNAc Metabolism). Thus, proteins co-clustered with the *agaS* and *agaA* genes were annotated as GalNAc specific and corresponding PTS systems were annotated as AgaPTS. (5) The EIIC proteins that co-clustered with the *agaS* but not the *agaA* genes were annotated as GalN-specific and corresponding PTS systems were annotated as GamPTS.

#### Prediction of a novel GlcNAc-specific transporter

An alternative ABC transporter for GlcNAc was predicted in this work and named NgcABCD. This transporter was predicted by analysis of gene clusters. Thus, genes for putative ABC transport system were co-clustered together with the *nagKAB* genes in 15 genomes of *Bifidobacterium* spp. The following differences were found between the NgcABCD and the previously described ABC-transporter NgcEFG (Xiao et al., 2002). First, the *ngcEFG* operon encodes only the substrate-binding protein and two intermembrane proteins, whereas the *ngcABCD* operon encodes an additional protein, an ATP-binding protein. Second, the substrate-binding subunits of these systems belong to different protein families; NgcE is a member of the SBP_bac_8 family (Pfam: PF13416), whereas NgcA is a member of the SBP_bac_5 family (Pfam: PF00496).

#### Prediction of a non-orthologous replacement for the uridylyltransferase GalT

To predict non-orthologous displacement of these genes, we used an analysis of gene occurrence also known as “Phyletic patterns” (Osterman and Overbeek, 2003; Tatusov et al., 2003). For the prediction of the non-orthologous displacement of *galT*, two sets of genomes were selected. The first set included finished genomes having *galK* but not *galT*. The second set included finished genomes having both *galK* and *galT* (Table S8). Finished genomes were selected because draft genomes do not allow us to distinguish the actual absence of the gene in the genome or the location of the gene in an unsequenced part of the genome. Candidate functional analogs of GalT were identified as genes present in genomes having only *galK* but absent in genomes having *galKT* genes. For the analysis of gene co-occurrence, we used the “Phylogenetic Profiler for Single Genes” tool available at the IMG JGI web-resource (https://img.jgi.doe.gov/). For the phylogenetic profiling the algorithm “By Present/Absent Homologs” was applied with the following parameters, maximal *e*-value = 1e-10, minimal identity = 30%, minimal percentage of taxa with homologs = 50%, and minimal percentage of taxa without homologs = 50%.

All the found 62 candidates (Table S8) were filtered to exclude membrane, regulatory, and secreted proteins, and then their domain structure and chromosomal environment were analyzed. The gene *Amuc*_0031 in the *A. muciniphila* genome (named *galY*), here was considered as the best candidate because of the following. (1) Encoded protein belongs to the nucleotidyl transferase (NTP_transferase, Pfam: PF00483) family, members of that are able to transform phosphosugars onto nucleotide sugars (Jensen and Reeves, 1998). (2) Orthologs of this gene from the analyzed genomes form a single monophyletic branch on a phylogenetic maximum-likelihood tree (Figure S7). Analysis of the tree together with the gene occurrence patterns showed that all homologs found in genomes lacking *galT* form a monophyletic branch. (3) Genes, encoding orthologs for this protein, were clustered with the *galE* gene in 15 analyzed genomes as wall with the *galMP* operon in two analyzed genomes.

#### Prediction of a novel galactose-specific transporter

A new Gal-specific transporter was predicted based on co-clustering of its gene together with *galK* in the genomes of *Propionibacterium* spp. and with *galKT* in the genomes of *Streptomyces* spp. This transporter is member of the SSS family (sodium solute symporter, Pfam: PF00474). The predicted function was checked by an analysis of chromosomal context for the orthologs of this gene.

## Results

### Collecting the data on degradation of mucin glycans and utilization of derived monosaccharides

Mucin glycans are complex polysaccharides that contain five different monosaccharides, L-fucose (Fuc), D-galactose (Gal), N-acetyl-D-galactosamine (GalNAc), N-acetyl-D-glucosamine (GlcNAc), and N-acetylneuraminic acid (Neu5Ac), and can form various glycosidic bonds (Podolsky, 1985; Tailford et al., 2015). Evidently, degradation of such complex structures requires a large number of bacterial proteins interacting with the mucin glycans. Because of this complexity and the variability of mucin glycans, systematization of bacterial proteins for the degradation of mucin glycans became the first goal of this study.

All bacterial proteins involved in the degradation of mucin glycans were divided into two groups: (1) GHs, which split glycans to oligo- and monosaccharides as well separating glycans from mucin proteins, and (2) enzymes required for the catabolism of the derived monosaccharides. This division was made based on the following aspects of mucin glycan degradation by the HGM. First, splitting of glycans and catabolism of the derived monosaccharides are spatially separated; the first process occurs outside of the microbial cell, while the second process occurs in the cell cytoplasm. Such spatial separation is important for the metabolic modeling of HGM-host metabolism. Second, some HGM organisms have only glycan-cleaving hydrolases or only monosaccharide-catabolizing enzymes. For example, *Bacteroides thetaiotaomicron* encodes only sialidases that can release Neu5Ac from mucin glycans but not genes for the catabolism of this compound (Marcobal et al., 2011). On the other hand, *Clostridium difficile* encodes only genes for the catabolism of N-acetylneuraminic acid but not sialidases (Sebaihia et al., 2006). Such differentiation of the enzymes provides for the cross-talk of the HGM organisms, as was shown for *B. thetaiotaomicron* releasing Neu5Ac and *Salmonella typhimurium* consuming it (Ng et al., 2013). Thus, the distribution of the monosaccharide-releasing enzymes and monosaccharide catabolic pathways should be analyzed separately.

**Figure 1:**
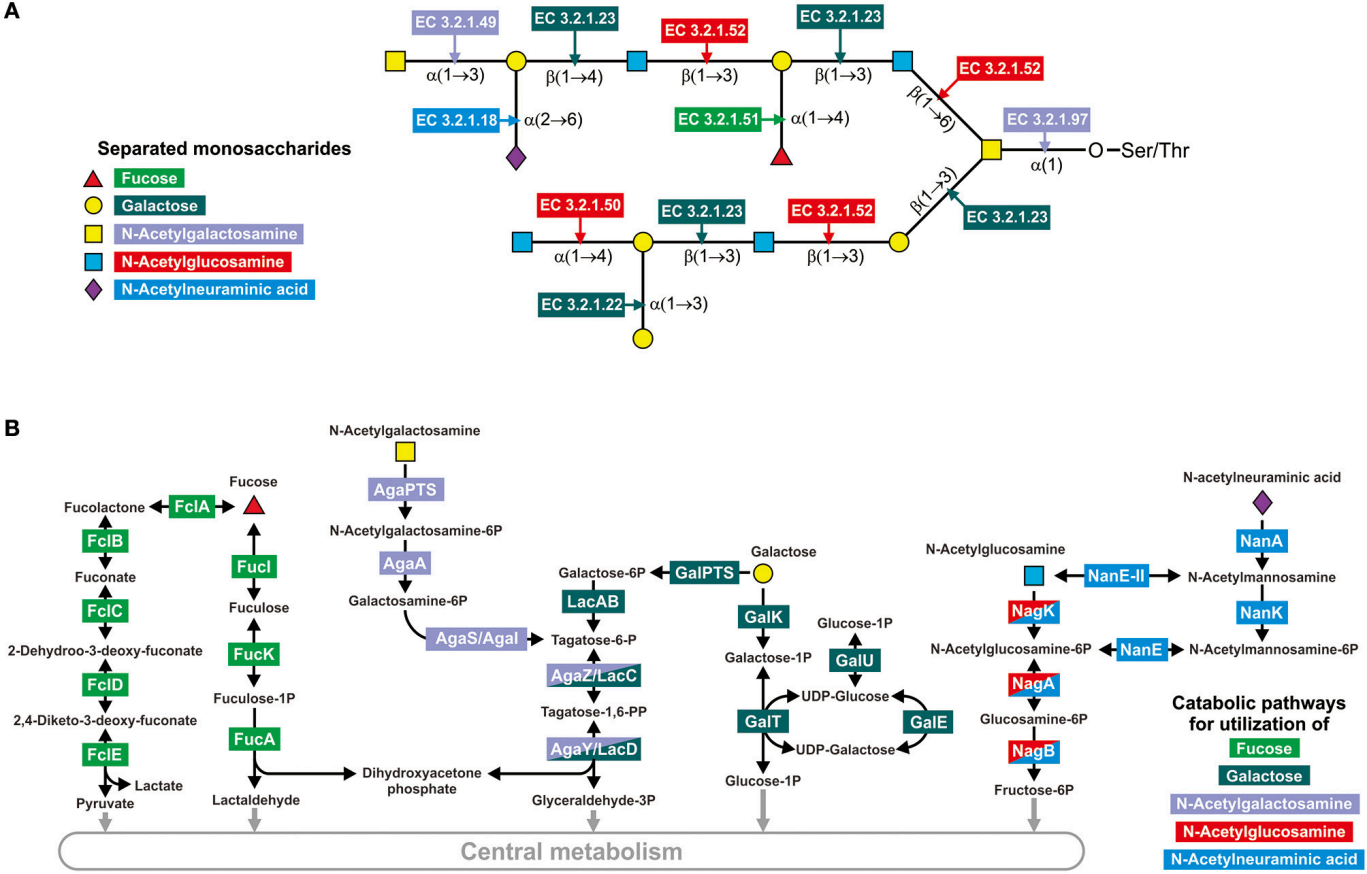
Enzymes involved in the degradation of mucin glycans. **(A)** Splitting of a hypothetical mucin glycan by GHs. The following enzymes are shown: EC 3.2.1.18, Neuraminidase; EC 3.2.1.22, α-galactosidase; EC 3.2.1.23, Beta-galactosidase; EC 3.2.1.49, α-N-acetylgalactosaminidase; EC 3.2.1.50, α-N-acetylglucosaminidase; EC 3.2.1.51, α-L-fucosidase; EC 3.2.1.52, β-N-hexosaminidase; EC 3.2.1.97, and Endo-α-N-acetylgalactosaminidase. **(B)** Known pathways for utilization of the derived monosaccharides. Protein abbreviations are shown; for the corresponding functions see Table S2 in the Supplementary Materials.

To identify all the GHs for mucin glycan degradation, we first identified all the glycosyl bonds previously detected in mucin glycans of the human intestine (Podolsky, 1985; Larsson et al., 2009; Tailford et al., 2015), which resulted in a collection of 21 different glycosyl bonds (Figure 1A and Table S9). Then, we searched for all the enzymes able to hydrolase such bonds using the KEGG (Kanehisa et al., 2012) and MetaCyc (Caspi et al., 2014) databases. Briefly, we searched the databases for both of the monosaccharides that form the bond after the reactions connected to this monosaccharide were filtered by EC number to identify all the glycoside hydrolases (i.e., enzymes with EC 3.2.-.-) for which this monosaccharide can be a substrate or product. After that, all the identified glycoside hydrolases were manually checked for the corresponding analyzed bond. Finally, we collected 9 types of GHs (Table S9). For further information on these enzymes, such as protein families and experimentally analyzed representatives, we carried out a search by EC number in the CAZy database (Cantarel et al., 2012).

Pathways for catabolism of the derived monosaccharides were identified here as sets of reactions necessary to convert the monosaccharides into any intermediates of central metabolism. The pathway data were extracted from the KEGG (Kanehisa et al., 2012) and MetaCyc (Caspi et al., 2014) databases as well as previous publications. For the two monosaccharides GalNAc and GlcNAc, only one pathway per monosaccharide has been described (Figure 1B). GalNAc is catabolized through tagatose 6-phosphate to glyceraldehyde 3-phosphate and dihydroxyacetone phosphate (Leyn et al., 2012; Bidart et al., 2014), whereas GlcNAc is converted into fructose 6-phosphate (Afzal et al., 2015a; Plumbridge, 2015; Uhde et al., 2016). For Fuc, Gal, and Neu5Ac, two alternative pathways for the catabolism of each monosaccharide have been described. Thus, Fuc may be catabolized through fuculose 1-phosphate to lactaldehyde and dihydroxyacetone phosphate or through fucolactone to lactate and pyruvate (Yew et al., 2006; Hobbs et al., 2013). For the genes encoding enzymes of the latter pathway, no four-letter abbreviations have been designated. Thus, for these genes, we introduced the designation *fclABCDE* (from fucolactone; the last letter corresponds to the order of catalyzed reactions in the pathway). Gal catabolism can also occur through two alternative pathways: through galactose 1-phosphate and UDP galactose (the Leloir pathway) (Bettenbrock and Alpert, 1998; Afzal et al., 2015b) or through galactose 6-phosphate and tagatose 6-phosphate (Zeng et al., 2010). The last two steps of the second pathway, phosphorylation and aldol splitting, are shared with the pathway for GalNAc catabolism. Neu5Ac is converted to fructose 6-phosphate by two pathways, through GlcNAc or GlcNAc 6-phosphate (Vimr et al., 2004; Brigham et al., 2009). Therefore, these pathways overlap with GlcNAc by two or three reactions, respectively (Figure 1B).

### Utilization of L-fucose

Intestinal mucin glycans contain Fuc moieties connected to Gal by α1,2-linkage as well to GlcNAc by α1,2-, α1,3-, or α1,4- linkages (Podolsky, 1985; Tailford et al., 2015). During the degradation of mucin glycans, these moieties can be removed by α-L-fucosidases (Katayama et al., 2004; Nagae et al., 2007; Ashida et al., 2009). α-L-fucosidases found in the analyzed genomes belong to three families: GH29, GH42, and GH95. At least one α-L-fucosidase was found in 131 genomes (Table S3). All of these genomes belong to only four phyla: Actinobacteria, Bacteroidetes, Firmicutes, and Verrucomicrobia. The largest number of genes encoding α-L-fucosidases were found in representatives of Firmicutes (*Lachnospiraceae bacterium* 3_1_57FAA_CT1, 16 genes) and Bacteroidetes (*Bacteroides coprophilus* DSM 18228, 14 genes).

Genes for both the alternative pathways for Fuc catabolism were found in the analyzed genomes. Thus, genes for the pathway through fuculose 1-phosphate (*fucIKA* genes) were found in the 125 genomes belonging to all studied bacterial phyla except Synergistetes and Tenericutes. The genes for the pathway through fucolactone (*fclABCDE*) were found in only 8 genomes, all belonging to Actinobacteria.

In total, three different Fuc-specific transport systems were found in the analyzed genomes, including two different permeases and one ABC transporter. The first permease, herein referred to as FucP1, was previously analyzed in *Escherichia coli* (Gunn et al., 1994) and predicted in the genomes of *Bacteroides* spp. (Hooper et al., 1999; Ravcheev et al., 2013). This transporter is highly distributed in the analyzed genomes, co-clustering with the *fuc* genes in 88 genomes and with the *fcl* genes in 4 genomes. An alternative Fuc permease named FucP2 belongs to the Sugar_tr family (Pfam: PF00083). The gene encoding this transporter is located inside the *fuc* gene cluster in the genomes of *Pediococcus acidilactici* and *Lactobacillus rhamnosus*. A third fucose transporter, the ABC-type one, was predicted in this study (see Prediction of Novel Fucose-Specific ABC Transport System). This transporter was found in 33 genomes belonging mostly to Actinobacteria and Clostridia.

**Figure 2:**
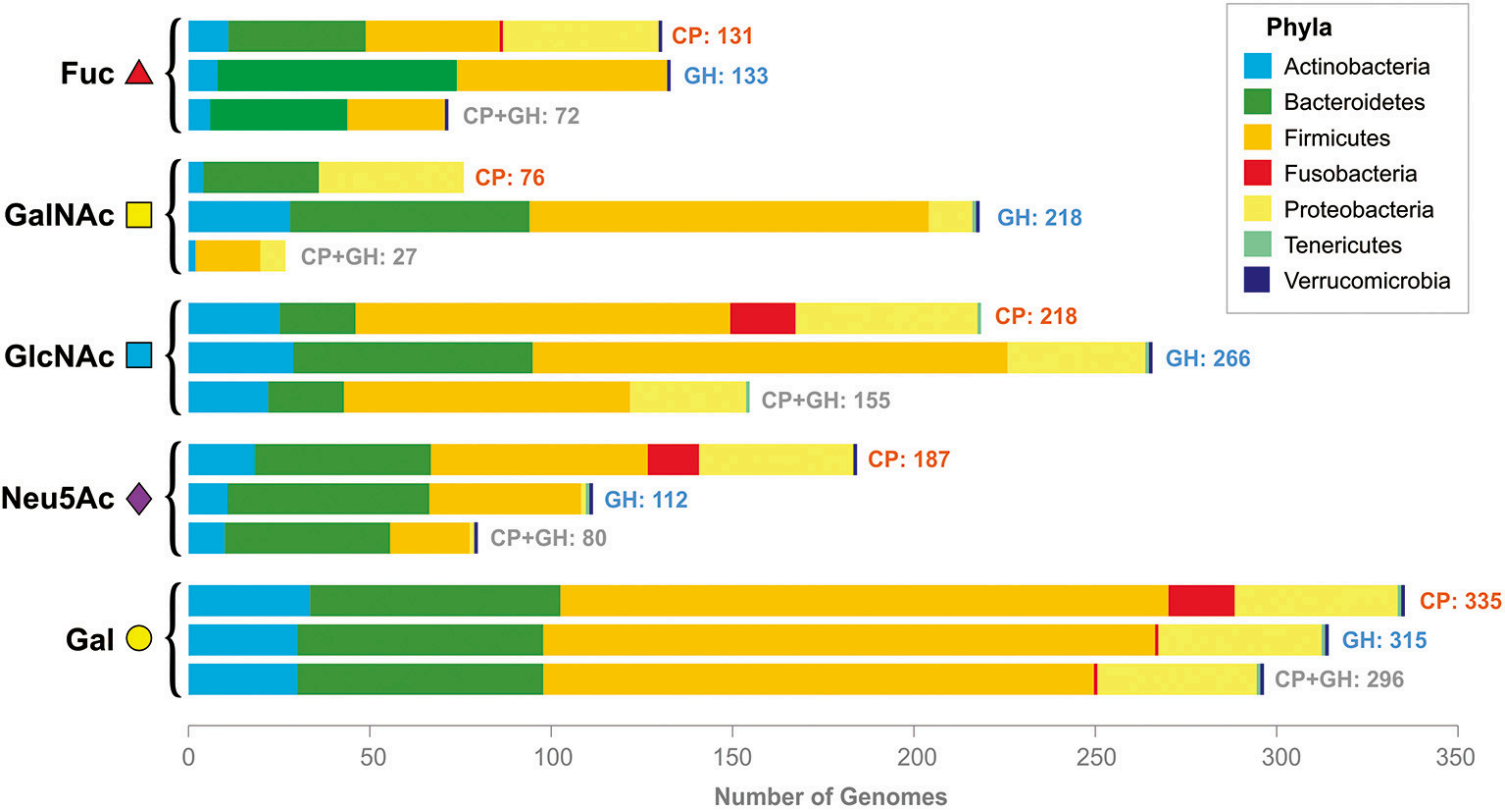
Distribution of the CPs and GHs for mucin glycan-derived monosaccharides in the analyzed genomes. Pathways and GHs are grouped according to the utilized monosaccharide. The bars “CP” correspond to the presence of catabolic pathways, the bars “GH” correspond to the presence of glycosyl hydrolases, the bars “CP+GH” correspond to the presence of GHs and pathways for this monosaccharide in the same genomes.

Generally, α-L-fucosidases were found in 131 analyzed genomes, whereas Fuc CPs were found in 133 genomes. Both α-fucosidases and CPs were found together in 72 analyzed genomes (Figure 2, Table S3).

### Utilization of N-acetyl-D-galactosamine

GalNAc plays a crucial role in mucin glycans, forming links with side-chain oxygen atoms of Ser/Thr residues in mucin peptide chains. This linkage between the glycan and peptide parts of mucin is mediated by various endo-α-N-acetylgalactosaminidases (Ashida et al., 2008; Koutsioulis et al., 2008; Kiyohara et al., 2012). Additionally, in intestinal mucin glycans, GalNAc moieties can be connected to Gal by α1,3-, or β1,4-linkage and to GalNAc by α1,6- or β1,3-linkage (Podolsky, 1985; Tailford et al., 2015). Release of GalNAc from mucin glycans can be mediated by β-N-hexosaminidases (Cabezas, 1989; Zwierz et al., 1999) and exo-α-N-acetylgalactosaminidases (Hoskins et al., 1997).

At least one GalNAc-releasing GH (GalNAc-GH) was found in 218 genomes (Table S4). No GalNAc-GHs were found in the genomes of Archaea or in the bacterial phyla Fusobacteria and Synergistetes. The maximal number of genes for the GalNAc-GHs was found in *Bacteroides* sp. 1_1_6 and *Bacteroides* sp. D22. Both of these organisms have 17 GalNAc-GH genes per genome. All exo-α-N-acetylgalactosaminidases in the analyzed genomes belong to the GH27 family. This type of GalNAc-GHs were found in only 13 genomes belonging to only four phyla: Actinobacteria, Bacteroidetes, Firmicutes, and Proteobacteria. Endo-α-N-acetylgalactosaminidases are also rarely represented in the analyzed genomes. These enzymes, belonging to the GH101 or GH129 families, were found in only 15 genomes from only two phyla: Actinobacteria and Firmicutes. In contrast, β-N-hexosaminidases were found in 217 genomes and in all bacterial phyla except Fusobacteria and Synergistetes. All the β-N-hexosaminidases found in the analyzed genomes belong to the GH3 or GH20 families.

GalNAc utilization pathway including *agaS* and *agaA* genes (see Annotation of Homologous Enzymes for GalNAc and GlcNAc Metabolism for Details on Annotation) was found in 76 analyzed genomes. Three different GalNAc-specific transport systems were found in the analyzed genomes, the PTS-type transporters AgaPTS and GnbPTS (see Annotation of the GalNAc-Specific Transporters for Details on Annotation), as well ABC-type transporter LnbABC. The GnbPTS is a transporter with multiple specificities and, in addition to GalNAc it can also transport oligosaccharides, lacto-N-biose (Galβ-1,3-GlcNAc), and galacto-N-biose (Galβ-1,3-GalNAc) (Bidart et al., 2014). Both lacto-N-biose and galacto-N-biose contain the glycosyl bonds found in intestinal mucin glycans (Table S9). Thus, GnbPTS was included in the three analyzed pathways as GalNAc, GlcNAc, and Gal catabolism. Lacto- and galacto-N-biose are then hydrolyzed by intracellular GH GnbG. Formally, GnbG is a GH, but because of its intracellular localization, it is considered to be a part of the GalNAc, GlcNAc and Gal CPs. The LnbABC transport system also transports lacto- and galacto-N-biose that afterwards hydrolyzed by intracellular phosphorylase LnbP (Nishimoto and Kitaoka, 2007). As with GamPTS-GamB, the LnbABC-LnbP system was considered to be a part of the GalNAc, GlcNAc and Gal CPs.

Overall, the GalNAc CP was found in the 76 analyzed genomes belonging to the phyla Actinobacteria, Firmicutes, and Proteobacteria (Figure 2, Table S4). Distribution of the GalNAc-releasing GHs is much broader; these enzymes were found in 218 genomes. Both GHs and CPs were found in only 27 of analyzed genomes.

### Utilization of N-acetyl-D-glucosamine

Intestinal mucin glycans contain GlcNAc moieties that form various glycosyl bonds, such as α1,4-, and β1,3-linkages with Gal and β1,3-, and β1,6-linkages with GalNAc (Podolsky, 1985; Tailford et al., 2015). The α-linkages are hydrolyzed by α-N-acetylglucosaminidases (Shimada et al., 2015), whereas the β-linkages with Gal or GalNAc are hydrolyzed by β-N-hexosaminidases (see Utilization of N-Acetyl-D-Galactosamine).

At least one GlcNAc-releasing GH (GlcNAc-GH) was found in 257 genomes (Table S5). The maximal numbers of the genes encoding GlcNAc-GHs were found in *Bacteroides* sp. 1_1_14 (23 genes) and *B. thetaiotaomicron* (22 genes). In addition to β-N-hexosaminidases (see Utilization of N-acetyl-D-Galactosamine), α-N-acetylglucosaminidases (GH89 family) were found in 60 genomes (Table S5). All genomes in which α-N-acetylglucosaminidase genes were found belong to the phyla Actinobacteria, Bacteroidetes, Firmicutes, Proteobacteria, and Verrucomicrobia.

The GlcNAc CP (Yang et al., 2006; Plumbridge, 2015) was found in 218 of the analyzed genomes (Table S5). This pathway is broadly distributed among analyzed taxa and is absent only in Archaea and the bacterial phyla Synergistetes and Verrucomicrobia. Various GlcNAc-specific transport systems were identified in the analyzed genomes. These are the PTSs NagE (Plumbridge et al., 1993; Plumbridge, 2015) and GnbPTS (see Utilization of N-Acetyl-D-Galactosamine), the ABC transport systems NgcEFG (Xiao et al., 2002), NgcABCD (predicted in this work, see Prediction of a Novel GlcNAc-Specific Transporter) and LnbABC (Nishimoto and Kitaoka, 2007), and the permease NagP (Ravcheev et al., 2013).

The GlcNAc moieties of intestinal mucin glycans can be sulfated. This sulfation possibly protects these glycans from degradation by the HGM and is correlated with health and disease status of the host organism (Tobisawa et al., 2010; Boltin et al., 2013). Only specific microbial species are able to remove sulfate groups to make mucin glycans available for themselves or to other microbes (Smalley et al., 1994; Robertson and Wright, 1997; Jansen et al., 1999; Wright et al., 2000; Robinson et al., 2012). Among the analyzed organisms, genes for mucin-desulfating sulfatase (GlcNAc-6-sulfatase) were found only in 42 genomes from Bacteroidetes phyla and in the genome of *A. muciniphila* (Table S5). Notably, more than half of these genes are chromosomally clustered with the *nagKP* operon or with genes for β-N-hexosaminidases (Figure S8).

Overall, GlcNAc CP was found in 218 analyzed genomes, whereas GlcNAc-GHs were found in 266 genomes. Both the pathway and the GHs were found in 155 analyzed genomes (Figure 2, Table S5).

### Utilization of N-acetyl-D-neuraminic acid

Neu5Ac is commonly found in the terminal location of intestinal mucin glycans (Johansson et al., 2011; Mcguckin et al., 2011), forming α2,3-linkages with Gal and α2,6-linkages with Gal or GalNAc (Tailford et al., 2015). These bonds can be hydrolyzed by sialidases (Juge et al., 2016). Sialidases, all belonging to the GH33 family, were found in 112 analyzed genomes. Sialidases were found in the genomes of all bacterial phyla except Fusobacteria and Synergistetes. The maximal number of sialidase-encoding genes was found in *Bacteroides fragilis* 638R and *Bacteroides* sp. D22 genomes (7 genes per genome).

Two CPs for Neu5Ac have been described previously (Vimr et al., 2004; Brigham et al., 2009). These pathways can be distinguished by the sugar epimerases; the first pathway is characterized by the presence of N-acetylmannosamine-6-phosphate 2-epimerase (NanE), whereas the second pathway is characterized by the presence of N-acetylglucosamine 2-epimerase (NanE-II) (Figure 1B). These epimerases are homologous to each other, and to distinguish them, we constructed a maximum-likelihood phylogenetic tree (Figure S9). Nonetheless, NanE and NanE-II are homologs; they form clearly distinguishable monophyletic branches on the tree. Thus, the first Neu5Ac CP, with the NanE enzyme, was found in 131 analyzed genomes, belonging to the phyla Actinobacteria, Firmicutes, Fusobacteria, and Proteobacteria. The second pathway, with the NanE-II enzyme, was found in 56 genomes. Most of these genomes are Bacteroidetes, only 3 of them belong to Firmicutes, and *A. muciniphila* is a representative of Verrucomicrobia.

The CPs were found in 187 analyzed genomes, belonging to all bacterial phyla with the exception of Synergistetes and Tenericutes. For Neu5Ac, various types of transporters have been previously described (Thomas, 2016). In the analyzed genomes, we identified the following Neu5Ac transporters: an MFS-type transporter (NanT), a sodium solute symporter (NanX), two ABC transport systems (NanABC and NanABC2), and a TRAP transport system (NeuT) (Table S7). No novel genes for Neu5Ac transport or catabolism were predicted.

The Neu5Ac CPs were found in 189 analyzed genomes, while both the pathways and the sialidases were found in only 80 analyzed genomes.

### Utilization of D-galactose

In human intestinal mucin glycans, Gal can form α1,3-linkages with other Gal, β1,3-linkages with GalNAc and GlcNAc, and β1,4-linkages with GlcNAc (Podolsky, 1985; Tailford et al., 2015). These linkages can be hydrolyzed by various α- (Wakinaka et al., 2013; Han et al., 2014; Reddy et al., 2016) and β-galactosidases (Husain, 2010; Park and Oh, 2010; Michlmayr and Kneifel, 2014; Solomon et al., 2015). At least one galactosidase gene was found in 310 analyzed genomes, belonging to all bacterial phyla except Synergistetes. The maximal numbers of galactosidase genes were found in *Bacteroides cellulosilyticus* DSM 14838 (56 genes) and *Bacteroides* sp. D2 (50 genes). The α-galactosidases found in the analyzed genomes belong to families GH4, GH27, GH36, and GH43, whereas the β-galactosidases belong to families GH2, GH35, and GH42.

For Gal utilization, two alternative pathways are possible. The Leloir pathway, in which Gal is utilized through UDP galactose (Bettenbrock and Alpert, 1998; Afzal et al., 2015b), was found in 335 analyzed genomes belonging to all bacterial phyla except Synergistetes. The 256 of these genomes contained the *galU* and *galT* genes for uridylyltransferases, whereas the 70 other genomes contained the *galY* gene, which was predicted to be a non-orthologs displacement of the *galT* (see Prediction of a Non-orthologs Replacement for the Uridylyltransferase GalT). The alternative pathway, the utilization of Gal through tagatose 6-phosphate (Zeng et al., 2010), was found in only 29 analyzed genomes, belonging to Actinobacteria and Firmicutes. Surprisingly, genes for the Leloir pathway were also found in all 29 of these genomes.

Four previously known Gal transporters were found in the analyzed genomes. These are galactose (Essenberg et al., 1997) and galactose/lactose (Luesink et al., 1998) permeases, here referred to as GalP1 and GalP2, respectively; the galactose/methyl galactoside ABC transport system Mgl (Weickert and Adhya, 1993); and the galactose-specific PTS (Zeng et al., 2012). Also, we predicted a new Gal-specific transporter from the SSS family (sodium solute symporter, Pfam: PF00474), here referred to as GalP3 (see Prediction of a Novel Galactose-Specific Transporter). The gene for this transporter was found to be co-clustered with the genes for the Leloir pathway in 20 analyzed genomes and with galactosidases in 16 analyzed genomes, that corroborate the predicted function. Additionally, Gal can be imported into the cell as part of lacto-N-biose and galacto-N-biose by the GnbPTS and/or LnbABC systems (see Utilization of N-Acetyl-D-Galactosamine and Utilization of N-Acetyl-D-Glucosamine).

Gal CPs were found in 355 analyzed genomes, whereas both Gal-GHs and the pathways were found in 296 genomes.

## Discussion

### Distribution of GHs and CPs in the analyzed HGM genomes

In this study, we analyzed the distribution of genes required for utilization of mucin glycans in 397 genomes of microbes found in the human gastrointestinal tract. The analyzed genes encode extracellular enzymes for cleavage of mucin glycans to monosaccharides as well as transport proteins and enzymes for subsequent utilization of the derived monosaccharides. These genes were conditionally divided into five groups, representing the five monosaccharides found in mucin glycans. Each of these monosaccharide-specific groups of genes can, in turn, be subdivided into extracellular GHs (Figure 1A) and genes for monosaccharide-utilizing CPs (Figure 1B). Analyzing the distribution of glycan-utilizing genes, we observed two general trends: (1) numerous genomes contain only CPs or only GHs and (2) pathways for different monosaccharides are distributed very differently from each other (Figure 2).

The presence of only CPs or only GHs has been previously described for the metabolism of Fuc (Pacheco et al., 2012; Conway and Cohen, 2015) or Neu5Ac (Ng et al., 2013) in the HGM. Nonetheless, such partial pathways have been previously described for only some HGM genomes. Here, our results demonstrated that such partial pathways are characteristic for the degradation of mucin glycans by the HGM. Thus, 339 (85%) of the analyzed genomes have a partial pathway for at least one of the mucin-derived monosaccharides. Generally, 726 partial pathways were found, including 312 cases with the presence of only CPs and 414 cases with the presence of only GHs (Table S10). Such a wide distribution of partial pathways indicates the existence of multiple potential feeding pathways for mucin glycan-derived monosaccharides in the HGM (see Possible Feeding Pathways for the Analyzed HGM Genomes).

A distinct distribution of pathways for utilization of the analyzed monosaccharides has not been described previously at the level of large microbial communities, such as the HGM. At the level of monosaccharide-specific CPs, sorting by the number of genomes where they were found produces the following sequence: Gal>GlcNAc>Neu5Ac>Fuc>GalNAc. A similar sequence was found for the GHs: Gal>GlcNAc>GalNAc>Fuc>Neu5Ac. The differences between these two sequences can be explained as follows. (1) GHs specific for Fuc and Neu5Ac were found in 72 and 80 genomes, respectively. Therefore, their switching places may be the result of a minor bias caused by the selection of the analyzed genomes. Because only genomes available in the HMP, KEGG and PubSEED databases were analyzed (see Approach, Tools, and Databases), the set of selected genomes is slightly biased. Thus, the set of analyzed genomes is enriched by genomes of Proteobacteria, especially by Enterobacteriales. (2) This difference also indicates that gastric mucin glycans are mainly neutral and that sialylation of them is quite rare (Rossez et al., 2012). (3) The higher number of genomes with GalNAc-GHs in comparison with Fuc- and Neu5Ac-GHs can be explained by the fact that the majority of GalNAc-GHs are β-hexosaminidases, which are enzymes specific to GalNAc and GlcNAc. This explanation is in good agreement with the large number of genomes having GlcNAc-specific CPs and/or GHs.

The different distributions of pathways for different monosaccharides may be due to their dissemination in nature, particularly in the human intestine. Therefore, in animals, Fuc and Neu5Ac are found mostly on terminal units of carbohydrate chains linked to proteins or lipids (Staudacher et al., 1999; Vimr, 2013; Pickard and Chervonsky, 2015). GalNAc is a slightly more disseminated since it is not only a terminal monosaccharide but also acts as a connecting link between the glycan and protein portions of N- and O-linked proteoglycans (Ashida et al., 2008; Koutsioulis et al., 2008; Kilcoyne et al., 2012; Kiyohara et al., 2012). Thus, in the human intestine, these three monosaccharides are parts of host-synthesized glycans or are derived from dietary components of animal origin. GlcNAc is much more broadly disseminated and, in addition to being found in glycoproteins, it can be found in heparin, chondroitin sulfate, hyaluronan, and various human milk oligosaccharides (HMOs) (Lamberg and Stoolmiller, 1974; Garrido et al., 2011). Gal is even more broadly disseminated than GlcNAc and can be found in both animal- or plant-synthesized polysaccharides. Thus, Gal is a building block of arabinogalactan, pectic galactan, and HMOs. As a component of side chains, Gal may be found in type II rhamnogalacturonan and as a terminal unit in α-mannans, galactomannan, xyloglucan, and xylan (Kunz et al., 2000; Mohnen, 2008; Garrido et al., 2011; Marcobal et al., 2011).

To confirm that the distribution of monosaccharide-utilizing pathways and monosaccharide-specific GHs reflects dissemination of these monosaccharides in nature, we analyzed data for these monosaccharides from the KEGG database. For each of the monosaccharides, two parameters were analyzed: (1) the number of reactions that the monosaccharide is involved in as a product or a substrate and (2) the number of glycans containing this monosaccharide (Figure S10). The numbers of reactions and glycans for the monosaccharides are in line with the distribution of CPs and GHs in the analyzed genomes. Thus, analysis of the numbers of reactions for each monosaccharide resulted in the following sequence: Gal>GlcNAc>Neu5Ac>Fuc>GalNAc, which is identical to the sequence for genomes with monosaccharide-specific CPs. For the number of glycans, the sequence is as follows: Gal>GlcNAc>Fuc>GalNAc>Neu5Ac, which is similar to the sequence for genomes with monosaccharide-specific GHs.

Examining the variation of the monosaccharide-specific pathways, we wondered how the combinations of the utilized monosaccharides varied across the HGM genomes. Because only CPs or only GHs were found in 85% of the genomes, combinations of CPs and GHs were analyzed separately. We used binary information regarding the distribution of pathways in 397 HGM genomes, i.e., the presence or absence of a CP or GH in a genome (Table S10). We investigated the 2^5^ = 32 possible patterns of the eight studied pathways. Only 22 (69%) and 19 (60%) of the 32 possible patterns were found for CPs and GHs, respectively. The most frequent pattern for CPs represents catabolism of only Gal and GlcNAc. This pattern was found in 59 analyzed genomes, including Bifidobacteriacea, various Firmicutes, and Fusobacteria. Other frequently observed CP patterns are as follows: (1) absence of CPs for any analyzed monosaccharides, (2) utilization of all monosaccharides except GalNAc, (3) utilization of Gal only, and (4) utilization of Gal and Neu5Ac only. The most frequent pattern for GHs is the presence of GHs specific to all five monosaccharides. This pattern was found in 81 genomes, including some Actinobacteria, multiple Bacteroidetes, Firmicutes (mostly belonging to the Lachnospiraceae family), and *A. muciniphila*. Other frequently observed GH patterns are as follows: (1) presence of GHs specific to GalNAc, GlcNAc, and Gal; (2) absence of GHs for any analyzed monosaccharides; (3) presence of only Gal-specific GHs; and (4) presence of only GlcNAc- and Gal-specific GHs.

Theoretically, the combination of the observed CP and GH patterns should result in 22^*^19 = 418 combined patterns; however, only 102 (24%) of them were actually observed, which indicates interdependence of GHs and CPs. This interdependence appears to be rather trivial because only non-digestible carbohydrates are available for HGM organisms, especially in the large intestine (Walker et al., 2011), so CPs are highly dependent on GHs. On the other hand, no significant correlations were observed between CP and GH patterns. Together with CP dependence on GH repertoire, this absence of correlations again indicates the possibility of intensive feeding by mucin-derived monosaccharides in the HGM.

A non-random character of the CP and GH distribution patterns was also confirmed by statistical Chi-squared testing. Such a testing was done for three types of distributions, CP patterns, GH patterns, and combined CP-GH patterns and resulted in the following output parameters. (1) For the CP patterns Chi-squared test results were X-squared = 727.51, degree of freedom *(DF)* = 31, and *p*-value < 2.2e-16. (2) For the GH patterns the test results were X-squared = 1303, *DF* = 31, and *p*-value < 2.2e-16. (3) For the combined CP-GH patterns the test results were X-squared = 10418, *DF* = 1023, and *p*-value < 2.2e-16. Thus, the tests showed that the distributions of all three pathways were non-random.

Surprisingly, the combined pattern corresponding to the most frequent GH and CP patterns was found in only three genomes: *Clostridium nexile* DSM 1787, *Lachnospiraceae bacterium* 2_1_46FAA, and *Ruminococcus lactaris* ATCC 29176. The combined pattern we observed most frequently was the absence of all analyzed GHs and CPs. This pattern was found in 28 genomes, belonging to Archaea, some Firmicutes, and Beta- and Epsilonproteobacteria. Other frequently observed combined patterns were as follows: (1) presence of GHs specific for GalNAc, GlcNAc, and Gal together with CPs for GlcNAc and Gal; (2) presence of GHs for all five monosaccharides together with utilization of all these monosaccharides except GalNAc; and (3) presence of GHs for all five monosaccharides together with utilization of Neu5Ac and Gal.

Taken together, an optimal strategy for glycan-utilizing HGM microorganisms includes (1) the presence of CPs specific to GlcNAc and Gal as the components of multiple host- and dietary-derived carbohydrates and (2) the presence of GHs specific to as large as possible a number of glycan-building monosaccharides.

### Possible feeding pathways for the analyzed HGM genomes

The presence of only CPs for a certain monosaccharide in one HGM organism and only GHs for this monosaccharide in another organism allows us to predict possible feeding pathways. Previously, such feeding pathways in the HGM have been found for Fuc (Pacheco et al., 2012; Conway and Cohen, 2015) and Neu5Ac (Ng et al., 2013). Here, we predicted multiple potential feeding pathways for all five monosaccharides forming mucin glycans. The 339 (85%) analyzed genomes demonstrated the presence of only CPs or only GHs for at least one monosaccharide; therefore, the majority of HGM organisms are involved in these feeding pathways.

Based on the presence or absence of CPs and GHs, each organism can be classified as a “donor” (having GHs but not CPs) or “acceptor” (having CPs but not GHs) in relation to a certain monosaccharide. Among the analyzed genomes, 181 (46%) organisms can only be “donors” while 103 (33%) organisms can only be “acceptors” for the studied monosaccharides (Table S10). Additionally, 55 (14%) organisms were classified as “mixed”, being “donors” for some monosaccharides and “acceptors” for others.

**Figure 3:**
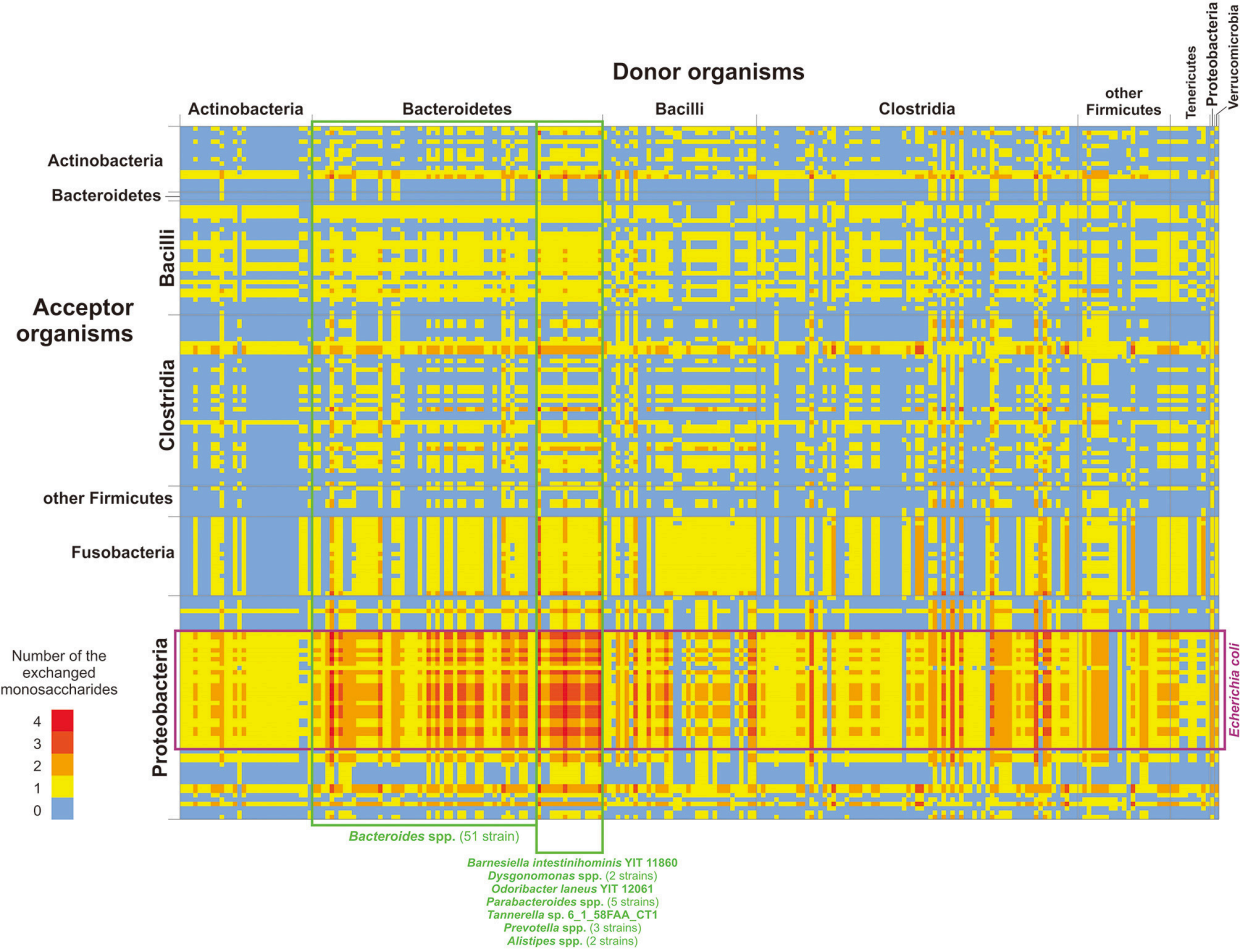
Distribution of monosaccharide donors and acceptors by taxa. Only genomes classified as “donors” (the horizontal axis) and “acceptors” (the vertical axis) are shown. The numbers of monosaccharides are shown in agreement with the color scale. For details on each pair of organisms and possibly exchanged monosaccharides, see Table S11.

In summary (Figure 3 and Tables S10, S11), data on possible feeding pathways demonstrate the following features of utilization of mucin-derived monosaccharides in the HGM: (1) larger number of “donors” relative to “acceptors” and (2) taxonomy-specific distribution of donors and acceptors.

Generally, 414 “donor” roles were found to be distributed among 236 genomes, whereas 312 “acceptor” roles were found to be distributed among 158 genomes. The significant predominance of “donors” reflects an adaptation of the analyzed organisms to the environment of the human intestine. No free monosaccharides are available in the large intestine (Walker et al., 2011), so the only way for HGM organisms to obtain monosaccharides is the cleavage of polysaccharides that are non-digestible for the host. Thus, HGM organisms should have GHs not only for monosaccharides that they can catabolize but also for all monosaccharides present in the available polysaccharides. Additionally, the appearance of a new “donor” role during evolution is much more probable than the appearance of a new “acceptor” role. With the appearance of a “donor,” only one new function should be acquired through horizontal gene transfer or duplication and neofunctionalization of the GH-encoding gene. Indeed, horizontal transfer has been previously demonstrated for genes encoding sialidase (Roggentin et al., 1993). In contrast, the appearance of a new “acceptor” requires horizontal transfer of multiple genes for enzymes and transporters. Because genes for CPs are not always encoded as a single locus (Leyn et al., 2012; Ravcheev et al., 2013; Rodionov et al., 2013; Khoroshkin et al., 2016), horizontal transfer of all genes required for the catabolism of a certain monosaccharide would be nearly impossible. Similarly, the appearance of a new CP through duplication and neofunctionalization requires the simultaneous change of specificities for multiple genes, which is also very unlikely.

The distribution of “donor” and “acceptor” roles among the analyzed genomes is taxon-specific (Figure 3). Thus, no “donors” were found among Fusobacteria, and no “acceptors” were found among Tenericutes and Verrumicrobia. Among Bacteroidetes, only 2 strains of *Bacteroides eggerthii* were identified as “acceptors,” whereas 62 strains from this group were identified as “donors.” The opposite is observed for Proteobacteria, for which only 9 genomes can be “donors” and 51 genomes can be “acceptors.” At the phylum level, the most intensive exchange of monosaccharides should be between “donor” Bacteroidetes and “acceptor” Proteobacteria, especially between different *Bacteroides* spp. various strains of *Escherichia coli* (Figure 3). Thereby, potential feeding pathways in the HGM are formed by the interaction of two factors, (1) evolutionary history of microbial taxa and (2) adaptation of HGM organisms to the intestinal environment.

### Specificity of the HGM organism to mucin glycans

Mucin glycans are complex polysaccharides characterized by a variety of monosaccharide building blocks and bonds between these monosaccharides (Martens et al., 2009; Koropatkin et al., 2012; Rossez et al., 2012; Johansson et al., 2015; Tailford et al., 2015). Tens of mucin glycan motifs and structures have been described to date. These structures vary in terms of both monosaccharides and glycosidic bonds (Podolsky, 1985; Rossez et al., 2012; Tailford et al., 2015). Furthermore, the analyzed genomes vary in the patterns of GHs able to cleave mucin glycans (Table S10). Thus, we proposed that the analyzed organisms should demonstrate some preferences for cleaved mucin glycans.

Data on the known structures of mucin glycans found in the human intestine were collected from the literature (Podolsky, 1985; Rossez et al., 2012), resulting in 56 different glycan structures (Figure S11 and Table S12). For each analyzed genome, the ability to cleave each glycan structure was predicted. Glycan was considered able to be cleaved by a certain organism if the GHs for all glycoside bonds in the glycan were found in the genome of the organism. Bonds between GalNAc and Ser/Thr residues of the mucin peptide were excluded from the analysis because this bond is cleaved by endo-α-N-acetylgalactosaminidases found in only 15 of the analyzed genomes (Table S4). On the basis of this prediction for each genome, the pattern of likely cleaved glycans was determined (Table S13). Among 397 analyzed genomes, 321 (81%) were able to cleave at least one of the glycans; generally, 20 different glycan-cleavage patterns were defined. It has previously been estimated that approximately 40% of bacteria have glycan-degrading enzymes (Arike and Hansson, 2016). Here, we demonstrated that, at least for the HGM microbes, this figure is actually at least 2-fold higher.

Based on the glycan-cleavage patterns, only 8 analyzed organisms are able to cleave all 56 glycan structures belonging to the phyla Bacteroidetes (*Bacteroides ovatus* SD CMC 3f, *Bacteroides* sp. 2_2_4, and *Bacteroides* sp. 3_1_23) and Firmicutes (*Clostridium perfringens* WAL-14572, 3 strains of *Lachnospiraceae bacterium*, and *Ruminococcus torques* ATCC 27756). The three most frequently observed patterns were the following. (1) Only Core 1 (Tailford et al., 2015) structures can be cleaved, i.e., no GHs except β-galactosidases are present. This pattern was found in 77 genomes belonging mostly to Lactobacillaceae, Ruminococcaceae and Enterobacteriales. (2) Glycans having only poly-lacto-N-biose but lacking any specific groups (Table S12) can be cleaved. These genomes have only GHs for the hydrolysis of β-Gal and β-GlcNAc bonds. This pattern was found in 56 genomes belonging mostly to Actinobacteria, Firmicutes and Enterobacteriales. (3) All glycans lacking α-GalNAc groups can be cleaved. These genomes have all GHs but not α-N-acetylgalactosaminidases. This pattern was found in 41 genomes belonging mostly to Bacteroidaceae.

**Figure 4:**
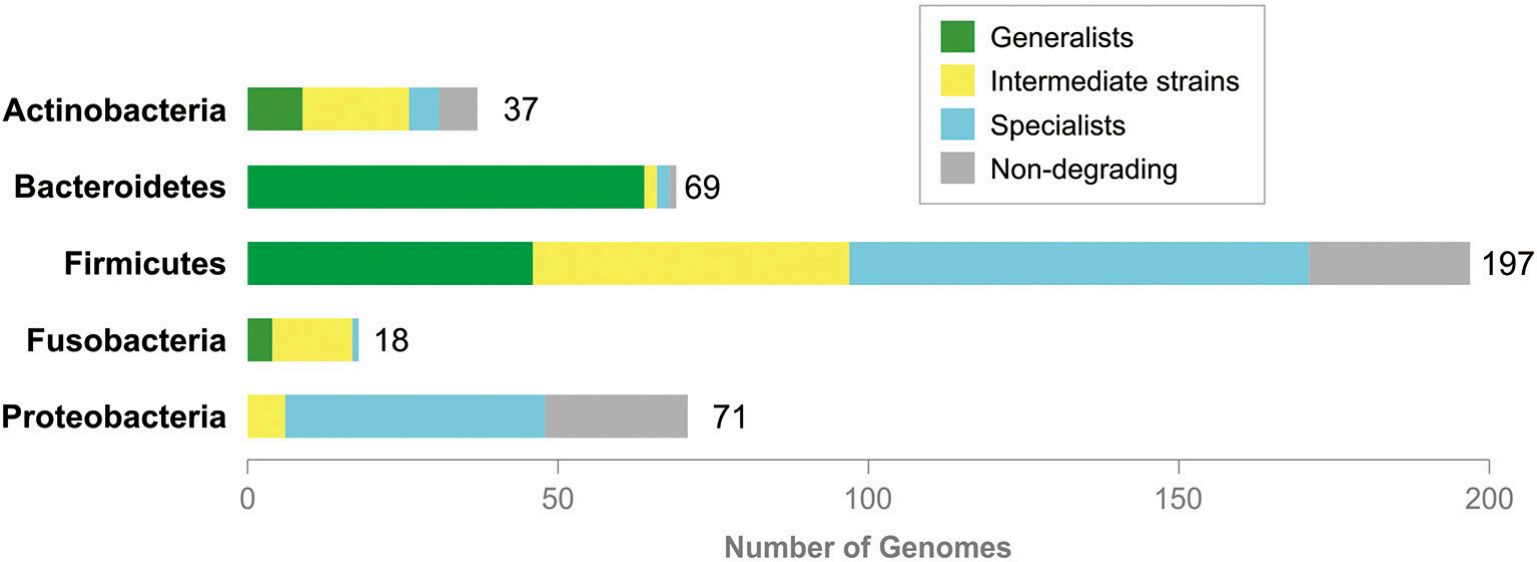
Generalists and specialists in degradation of mucin glycans. Total numbers of the analyzed genomes are shown right to tabs. No information is shown for Archaea (both analyzed organisms are non-degrading) and taxa with one analyzed genome, Synergistetes (non-degrading), Tenericutes (generalist), and Verrucomicrobia (generalist).

Based on the repertoire of possibly hydrolyzed mucin glycans, the analyzed genomes can be classified as generalists or specialists as follows. (1) Organisms predicted to degrade 1 to 3 glycans were classified as specialists. (2) Organisms predicted to degrade 21 to 56 glycans were classified as generalists. (3) Organisms predicted to degrade 5 to 13 glycans were classified as intermediate strains. Distribution of generalists, intermediates, and specialists was clearly taxon-specific (Figure 4 and Table S13). Thus, most of the analyzed Actinobacteria are intermediates. In this taxon, there are also some generalists and specialists, with the larger number of generalists than specialists. An overwhelming majority of Bacteroidetes are generalists, whereas intermediates and specialists are exceptional in this taxon. Proteobacteria, on the contrary, are mostly specialists and no one generalist was found in this taxon. Firmicutes contain almost equal numbers of generalists and intermediates and slightly more specialists than generalists or specialists.

**Figure 5:**
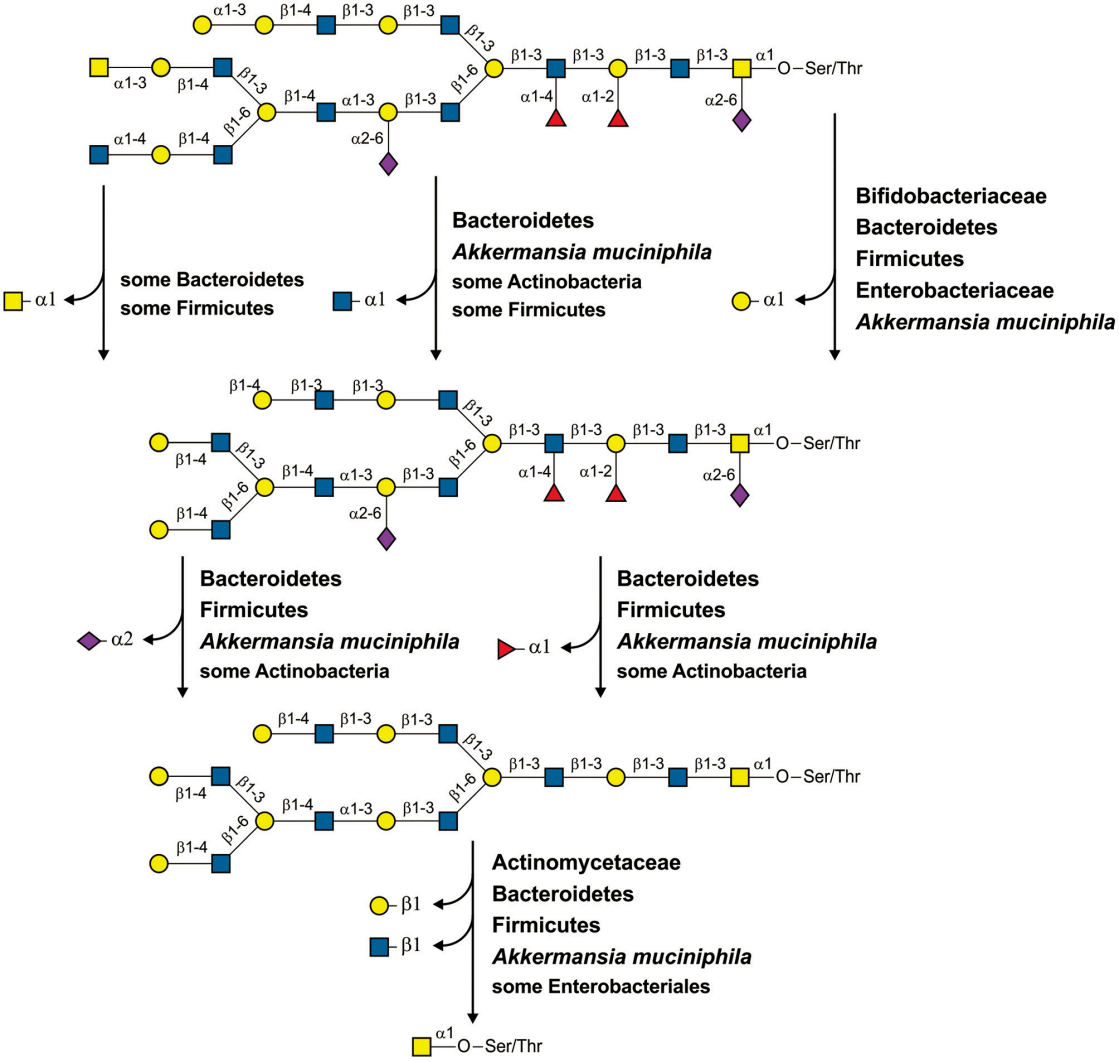
Cleavage of a hypothetical mucin glycan by a community of HGM inhabitants. For each reaction, the taxa with the corresponding specific GHs are shown.

The predicted glycan-cleavage patterns demonstrate some taxon-specific features. For example, for 66 genomes of glycan-cleaving Bacteroidetes, only 8 patterns were identified, whereas for 46 genomes of glycan-cleaving Proteobacteria, only 4 patterns were identified. Thus, the glycan-cleavage abilities of HGM organisms also depend on the evolutionary history of the taxon, though the correlation between these abilities and taxonomy is not so strong. On the other hand, mucin glycans can also be cleaved by multistrain communities in which each strain hydrolyzes a part of the glycosyl bonds (Figure 5).

An ability of HGM microbes to degrade different glycans has been previously determined using the GlyDeR pipeline (Eilam et al., 2014). This study included the analysis of 196 strains (Table S14) and 9 mucin glycans (namely, glycans 1, 11, 22, 24, 30, 44, 50, 51, and 54, see Figure S11) that overlapped with our work. Both studies predicted that 89 (45.4%) genomes cannot degrade mucin glycans. A total of 79 (40.3%) genomes were predicted to degradate mucin glycans only in the present study. For 22 genomes (11.2%), the glycan degradation was predicted by both studies, but the current study predicted more glycans. Six genomes (3.1%) were predicted by both studies, but GlyDeR pipeline predicted more glycans. Such a big differences between results of this work and GlyDeR-based predictions can be explained by the following reasons. (1) The GlyDeR pipeline is developed for a large-scale automated analysis, whereas this work was concentrated only on known intestinal mucin glycans. (2) In the GlyDeR pipeline, GHs are predicted by their similarity to proteins represented at the CAZy database (Cantarel et al., 2012) whereas we used as a query GHs extracted from multiple databases. In addition to CAZy, we used the UniProt (Magrane and Consortium, 2011), KEGG (Kanehisa et al., 2012), and MetaCyc (Caspi et al., 2014), as source of the query protein sequences. (3) The GlyDeR uses one-direction BLAST search for prediction of the GH specificities whereas here a bi-directional search together with an analysis of the protein domain structures and of the genome context was used. One-directional search often results in inexact functional annotations, especially for large protein families. For example, among GHs degrading human intestinal mucin glycans, there are two enzymes belonging to the GH42 family, the α-L-fucosidase and β-galactosidase (Table S2). Inaccurate annotations for these enzymes can sufficiently change the pattern of potentially degraded glycans. (4) The approach used in this study estimates an ability of the organism to degrade certain glycan as a Boolean function, answering to the question “Can this organism degrade this glycan?” by “yes/no.” In contrast, the GlyDeR pipeline estimates a specific score, and the “yes/no” answer depends on the threshold selected for this score. The GlyDeR scores for all the glycans analyzed in the both studies are <0.05 (Table S14), which is low. For example, the minimal non-zero GlyDeR score for lactose is a 0.3333 (Eilam et al., 2014). It appears that GlyDeR provides an estimation of the glycolytic potential for a large number of glycans, whereas an approach used in this study is better for detailed analysis of a small number of structurally related glycans.

**Figure 6:**
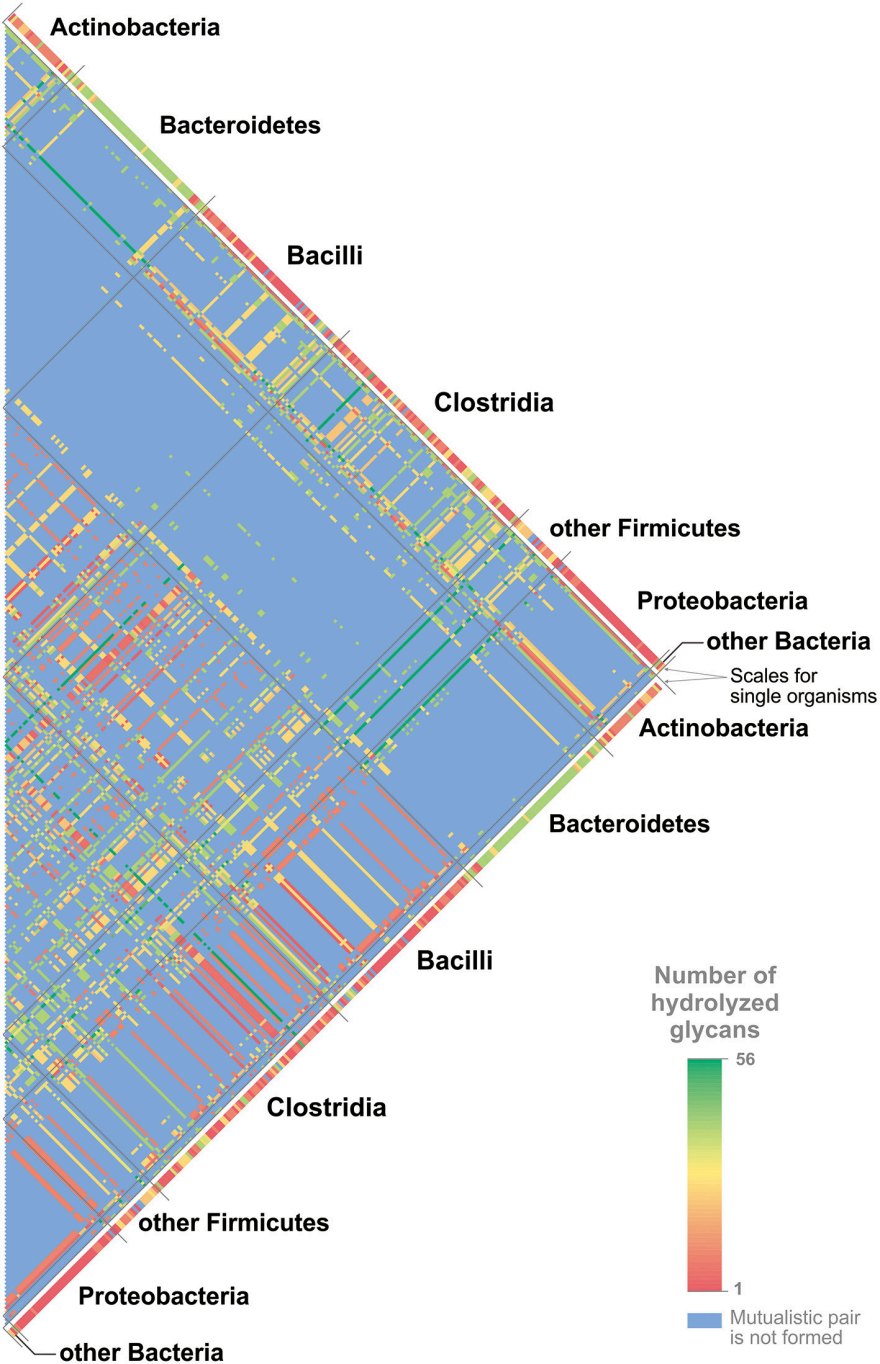
Mutualistic pairs for cleavage of mucin glycans. The numbers of cleaved glycans are shown in agreement with the color scale. The numbers of glycans cleaved by single organisms are shown for all genomes. For the pairs of organisms, the numbers of cleaved glycans are shown only for mutualistic pairs. For details on each mutualistic pair of organisms and cleaved glycans, see Table S15.

A minimal multistrain community should include two different strains. Here, we proposed the existence of mutualistic pairs of HGM organisms. We defined a predicted mutualistic pair as a pair of organisms that can cleave more mucin glycans than a union of the glycan-cleavage patterns of these two organisms. To predict such mutualistic pairs, we used the following procedure. (1) All possible pairs of the studied genomes were analyzed with the exception of pairs in which at least one of the organisms can cleave all 56 glycan structures (Figure S11). (2) For each of the analyzed pairs, two glycan-cleavage patterns were predicted. (3) The so-called “summary pattern” was defined as a union of the patterns for each organism in the pair. (4) The so-called “mutualistic pattern” was defined as a pattern predicted on the basis of the GHs present in both genomes. (5) For a pair of genomes, the common set of GHs was considered to be the sum of GHs found in each genome. (6) For the common set of GHs, the ability to cleave each glycan structure was predicted. (7) All the glycans able to be cleaved by the common set of GHs formed the “mutualistic pattern” for this pair of organisms. (8) If the “mutualistic pattern” contained glycans absent in the “summary pattern,” this pair of organisms was predicted to be a mutualistic one. In total, 325 (82%) analyzed genomes were able to form mutualistic pairs (Figure 6 and Table S14).

The predicted mutualistic pairs able to cleave all 56 glycan structures were considered to be highly beneficial. Thus, in agreement with predicted glycan-cleavage patterns, *Collinsella stercoris* DSM 13279 and *Clostridium spiroforme* DSM 1552 can cleave 27 different glycans each, *Eubacterium dolichum* DSM 3991 can cleave 9 glycans, and *Cedecea davisae* DSM 4568 cannot cleave any mucin glycans. Each of these genomes can form highly beneficial pairs with various *Bacteroides* spp. that alone can cleave 40–41 glycans. *Clostridium celatum* DSM 1785 has a glycan-cleavage pattern that includes 50 glycans. This genome can form highly beneficial pairs with *Bifidobacterium* spp. that can cleave 2–22 glycans, with *Lactobacillus* spp. that can cleave 0–22 glycans, and with Enterobacteriaceae that can cleave 0–9 glycans. All highly beneficial pairs described are organized in a similar manner. The five strains listed above are distantly related to each other, and three of them, *C. celatum, C. davisae*, and *C. spiroforme*, are pathogens (Akinosoglou et al., 2012; Papatheodorou et al., 2012; Agergaard et al., 2016), whereas no data about the pathogenicity of *C. stercoris* and *E. dolichum* were found. Each of these five strains forms highly beneficial pairs with a large number of organisms closely related to each other and highly represented in healthy HGM (Eckburg et al., 2005; Goodman et al., 2011; Walker et al., 2011; Graf et al., 2015). Based on these features of highly beneficial pairs, we proposed that these five organisms can be harmful to human health not only due to pathogenicity itself but also because they can greatly increase the ability of the HGM to forage the host mucus layer.

The idea of mutualistic pairs is quite attractive but rather speculative at this stage, as it requires additional support, such as ecological-statistical testing of its relevance. One would need to test how many of such pairs co-occur in actual human gut samples compared to a random co-occurrence model. However, such analysis is associated with some challenges. For instance, the HGM taxonomical structure significantly varies depending on host genetics, age, geography, lifestyle, and diet (Kurokawa et al., 2007; Clemente et al., 2012; Yatsunenko et al., 2012; Suzuki and Worobey, 2014; Allais et al., 2015). For example, only 75 microbial species were found in more than 50% of individuals (Qin et al., 2010). Second, HGM is characterized by functional redundancy, namely the same functions can be conferred by multiple bacteria, both closely or distantly related to each other (Moya and Ferrer, 2016). The predicted mutualistic pairs illustrate this redundancy. For example, *C. stercoris* was predicted to form mutualistic pairs, i.e., having an identical sets of mucin glycans degraded by the pair, with such a distantly related organisms as *A. muciniphila, B. thetaiotaomicron, C. nexile*, and *Streptomyces* sp. HGB0020 (Table S15). Finally, the results of the testing may be biased because of the closely related organisms that can be present in the HGM. For example, *C. stercoris* can form identical predicted mutualistic pairs with the 43 different *Bacteroides* spp. (Table S15). If different, but closely related to each other, *Bacteroides* spp. will be present in samples from different individuals, this testing will not demonstrate a co-occurrence of *C. stercoris* with any of these strains in comparison with a random co-occurrence. Taken together, idea of the mutualistic pairs in the HGM is a perspective area for further studies.

### Unresolved problems and possible solutions

This study resulted in the prediction of a number of novel genes involved in utilization of human mucin glycans. Nonetheless, some problems related to monosaccharide utilization remain unresolved (Tables S3–S8). These problems are the absence of one or two steps of certain CPs as well as the absence of known transporters in the presence of a corresponding CP. At least one such problem was detected for 90 (23%) of the analyzed genomes. The most frequently observed problems are as follows: (1) the absence of known Gal transporters in 30 genomes, mostly Firmicutes; (2) the absence of L-fuculose phosphate aldolase in 23 genomes belonging to Bacteroidetes, Clostridia, and some Actinobacteria; (3) the absence of L-fuculokinase in 19 genomes belonging to Bacteroidetes, Clostridia, and some Actinobacteria; and (4) the absence of galactose kinase, which was observed in 7 genomes of Firmicutes. Other problems have been observed in 1–6 analyzed genomes.

These unresolved problems can be explained by three non-exclusive hypotheses: (1) the incompleteness of genome sequences, (2) non-orthologous replacements for enzymes and transporters, and (3) the existence of alternative reactions and pathways. A total of 326 (82%) of the analyzed genomes have draft status, and some genes for the transport and utilization of monosaccharides may thus be absent from the current version of the genome. Indeed, 77 genomes with absent genes have draft status. Therefore, obtaining the finished genomes for the studied organisms will help us to fill the gaps in the incomplete pathways.

The problem of pathway incompleteness is only partially resolved by the finished versions of the analyzed genomes because incomplete pathways were also found in 13 finished genomes. For example, the finished genome of *Clostridium difficile* NAP07 lacks genes for Gal- and GalNAc-specific transporters, as well as for galactose kinase. These gaps may be filled by prediction or experimental identification of non-orthologous replacements, namely, genes that are not orthologs of the previously known genes but have the same functions. Such replacements have been previously described for the analyzed monosaccharide-utilization pathways. Thus, pairs of non-orthologs proteins were known for galactosamine-6-phosphate isomerase (AgaS and AgaI), glucosamine-6-phosphate deaminase (NagB1 and NagB2), and N-acetylglucosamine kinase (NagK1 and NagK2). Moreover, in this study, we predicted 4 non-orthologous replacements for enzymes (FclA2, FclD2, FclE2, and GalY) and 4 non-orthologous replacements for transporters (FucABC, FucP2, GalP3, and NgcABCD). The idea of non-orthologous replacement is very promising because these replacements can be found with computational methods alone.

Another possible way to resolve problems with incomplete pathways is the prediction of alternative reactions or pathways. Unlike the case of non-orthologous displacements, here, we should predict genes with previously unknown functions, but involved into metabolism of analyzed compounds. For example, catabolism of Fuc, Neu5Ac, and Gal is possible via two different pathways for each of these compounds (Figure 1B). The prediction of novel reactions in pathways is usually more difficult than the detection of non-orthologous displacements but is also possible using only computational methods. For example, an alternative pathway for the biosynthesis of menaquinone was discovered using comparative genomics techniques (Hiratsuka et al., 2008), and gaps in this pathway have subsequently been filled by computational analysis (Ravcheev and Thiele, 2016).

Taken together, all the remaining problems may be resolved using comparative genomics-based analysis. The availability of an increasing number of microbial genomes as well as completion of existing genome sequences will provide significant opportunities for the computational analyses of these microbes and the resolution of the described problems.

### Concluding remarks and future plans

This study included a comprehensive computational analysis of the degradation of mucin glycans by HGM microorganisms. In addition to novel functional annotations, which are standard for comparative genomics studies, this analysis also predicted commensal interactions between different microbes, specificity of the HGM strains to various types of glycans, and mutualistic inter-strain interactions at the level of mucin glycan degradation. These results demonstrate the efficiency of the selected approach: the analysis of individual genomes for members of the microbial community.

Although this study significantly improved our understanding of the HGM and its interactions with the host, there is still much to be discovered in this field. The areas lacking evidence indicate the future directions for the analysis of the HGM as it relates to mucin glycan degradation. The first two future directions, which are defined by unresolved problems (see Unresolved Problems and Possible Solutions), are to update the metabolic reconstructions with full versions of all analyzed genomes and to fill in the remaining gaps in the studied metabolic pathways. The third future direction is to expand the reconstructed metabolic, transport, and possible feeding pathways to novel genomes; this is aimed at the growing number of microbial genomes, including members of the HGM community. Mucin glycans are not the only glycans that can be degraded by HGM organisms. Thus, the fourth future direction is pathway reconstruction for the degradation of dietary-derived glycans.

The HGM has been intensively studied in relation to its impact on human health, and more than 50 human diseases have been shown to be associated with HGM alterations (Potgieter et al., 2015). Nonetheless, the use of HGM taxonomical composition as a diagnostic tool is currently hampered by the high variability of the HGM depending on various factors (Kurokawa et al., 2007; Clemente et al., 2012; Yatsunenko et al., 2012; Suzuki and Worobey, 2014; Allais et al., 2015) and by functional redundancy of the HGM. As it relates to the HGM, functional redundancy is defined as the ability of multiple bacteria, both closely and distantly related to each other, to implement the same metabolic functions (Moya and Ferrer, 2016). On the other hand, more than a dozen HGM-produced metabolites are associated with various human diseases (Potgieter et al., 2015), indicating possible associations between human health and the presence or absence of certain metabolic pathways in the HGM. Therefore, functional redundancy may provide a possible approach for the use of the HGM to distinguish health states of an individual. Indeed, a comparison of metagenomics data from healthy or diseased subjects may highlight HGM genes that are associated with disease. Because accurate functional annotation of all the genes in each metagenome is extremely time-consuming and costly, it makes sense to compare different microbiomes for the presence or absence of particular metabolic pathways that are already annotated and possibly associated with a health state. Because the state of the intestinal mucus layer is closely associated with human health (Png et al., 2010; Johansson et al., 2013; Cockburn and Koropatkin, 2016; Desai et al., 2016), HGM genes involved in the degradation of mucin glycans are perfect candidates to be tested for associations with health and disease. Thus, the fifth future direction is an analysis of the presence or absence of the analyzed genes in health and disease HGM metagenomes.

Computational modeling of metabolism (Palsson, 2006; Orth et al., 2010) may be used for elucidation of mucin glycan degradation by HGM organisms. Previously, genome-based models have been published for single representatives of the HGM (Thiele et al., 2005, 2011, 2012; Orth et al., 2011; Heinken et al., 2014) as well as for whole HGM communities (Levy and Borenstein, 2013; Bauer et al., 2015; Heinken and Thiele, 2015; Noecker et al., 2016; Shashkova et al., 2016; Magnúsdóttir et al., 2017). Additionally, computational models for human metabolism are also available (Thiele et al., 2013b; Mardinoglu et al., 2014), and host-microbial metabolic interactions have been modeled (Heinken et al., 2013, 2016; Thiele et al., 2013a; Shoaie and Nielsen, 2014; Levy et al., 2015). Thus, the final future direction is to update the existing HGM and host-HGM metabolic models of reactions for all the pathways reconstructed in this study. Such an update would improve the existing models and would improve our understanding of the interaction between humans and their microbiome.

## Author contributions

DR and IT conceived of and designed the research project and wrote the manuscript. DR performed the genomic analysis of the pathways for utilization of mucin glycans. All authors read and approved the final manuscript.

### Conflict of interest statement

The authors declare that the research was conducted in the absence of any commercial or financial relationships that could be construed as a potential conflict of interest. The reviewer WY and handling Editor declared their shared affiliation.
