# Supplementary figures and images for: Comparative Genomic Analysis of the Human Gut Microbiome Reveals a Broad Distribution of Metabolic Pathways for the Degradation of Host-Synthetized Mucin Glycans and Utilization of Mucin-Derived Monosaccharides
Source: Front Genet. 2017 Aug 29;8:111. doi: 10.3389/fgene.2017.00111 (PMC5583593; doi:10.3389/fgene.2017.00111)

Genome names are shown in brackets.

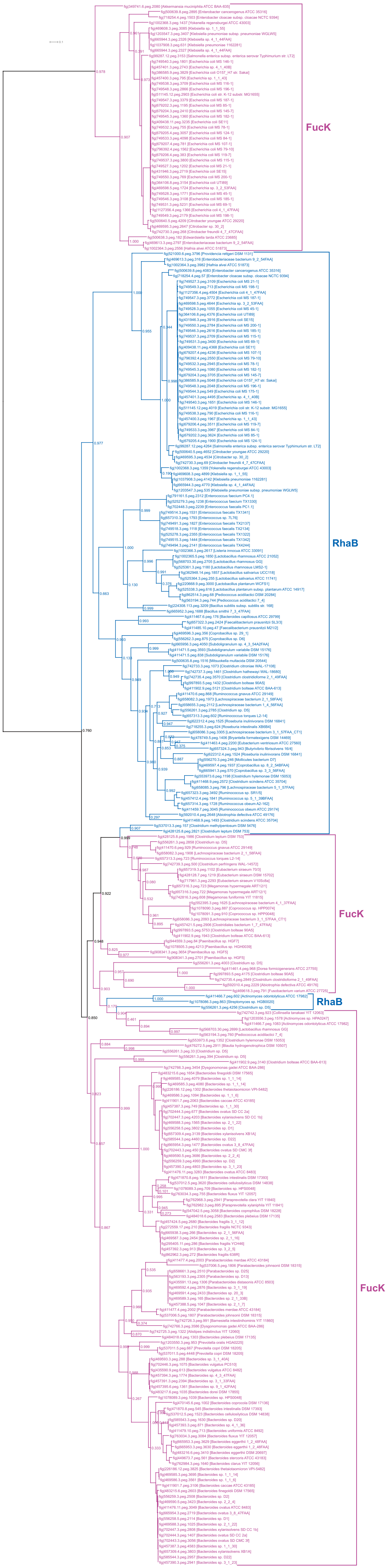

Supplement: Supplementary file 17 [file Image1.PDF]

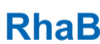

Supplement: Supplementary file 18 [file Image2.pdf]

© 2006 The Authors  
Journal compilation © 2006 Blackwell Publishing Ltd

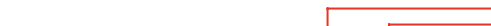

Supplement: Supplementary file 21 [file Image5.PDF]
