# Supplementary material for: Comparative Genomic Analysis of the Human Gut Microbiome Reveals a Broad Distribution of Metabolic Pathways for the Degradation of Host-Synthetized Mucin Glycans and Utilization of Mucin-Derived Monosaccharides
Source: Front Genet. 2017 Aug 29;8:111. doi: 10.3389/fgene.2017.00111 (PMC5583593; doi:10.3389/fgene.2017.00111)

Figure S3. Maximum-likelihood tree for the FclA and Fcld proteins. Functions are shown by colors. The SEED identifiers for proteins are shown; for their sequences, see the file Sequences S1 in the Supplementary materials. For the experimentally analyzed proteins (shown in bold) locus_tag identifiers are shown. Genome names are shown in brackets.

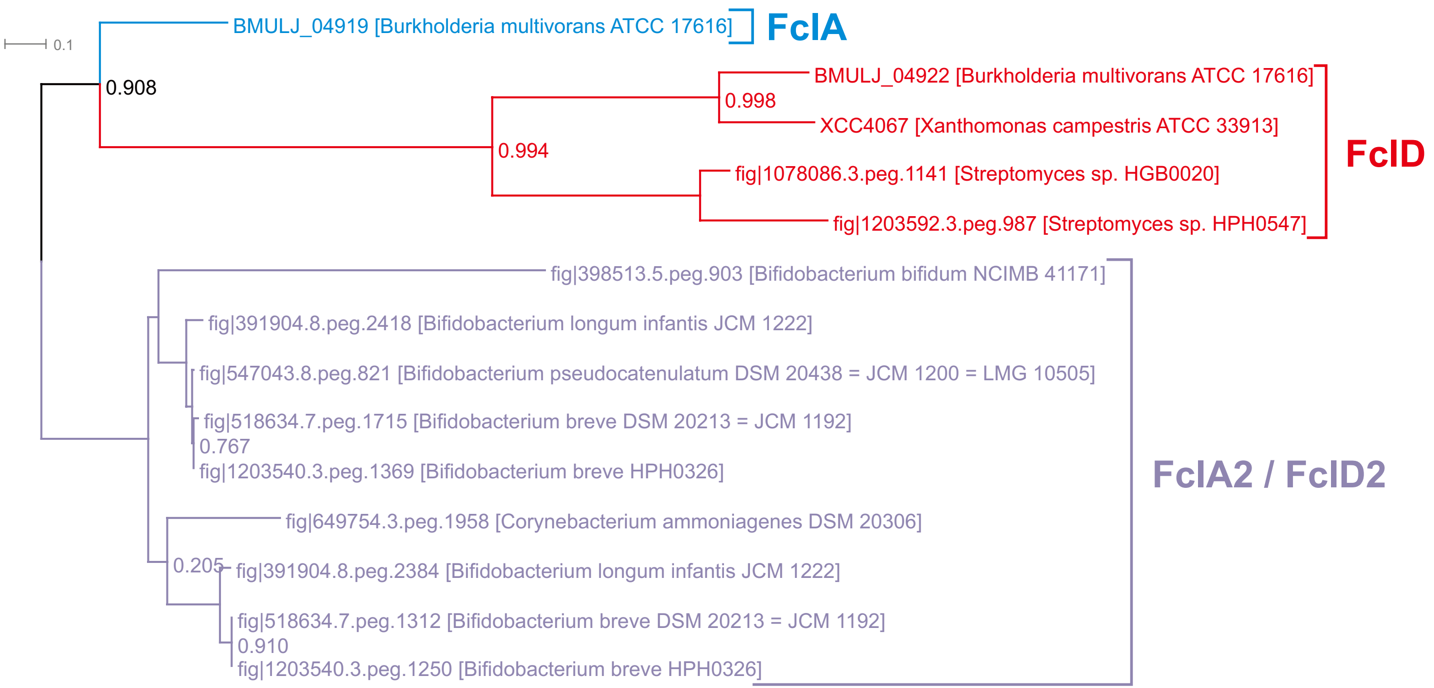

Supplement: Supplementary file 19 [file Image3.PDF]
