# Supplementary material for: Comparative Genomic Analysis of the Human Gut Microbiome Reveals a Broad Distribution of Metabolic Pathways for the Degradation of Host-Synthetized Mucin Glycans and Utilization of Mucin-Derived Monosaccharides
Source: Front Genet. 2017 Aug 29;8:111. doi: 10.3389/fgene.2017.00111 (PMC5583593; doi:10.3389/fgene.2017.00111)

Figure S8. Maximum-likelihood tree for the Gns (N-acetyl-D-glucosamine specific mucin-desulfating sulfatase) proteins. Predicted sulfatase from *Bacteroides ovatus* SD CC 2a used as outgroup is shown by gray. Genes chromosomally clustered with the *nagKP* operon are shown by green, genes chromosomally clustered with the gene(s) for beta-hexosaminidase are shown by blue. The SEED identifiers for proteins are shown; for their sequences, see the file Sequences S1 in the Supplementary materials. Genome names are shown in brackets.

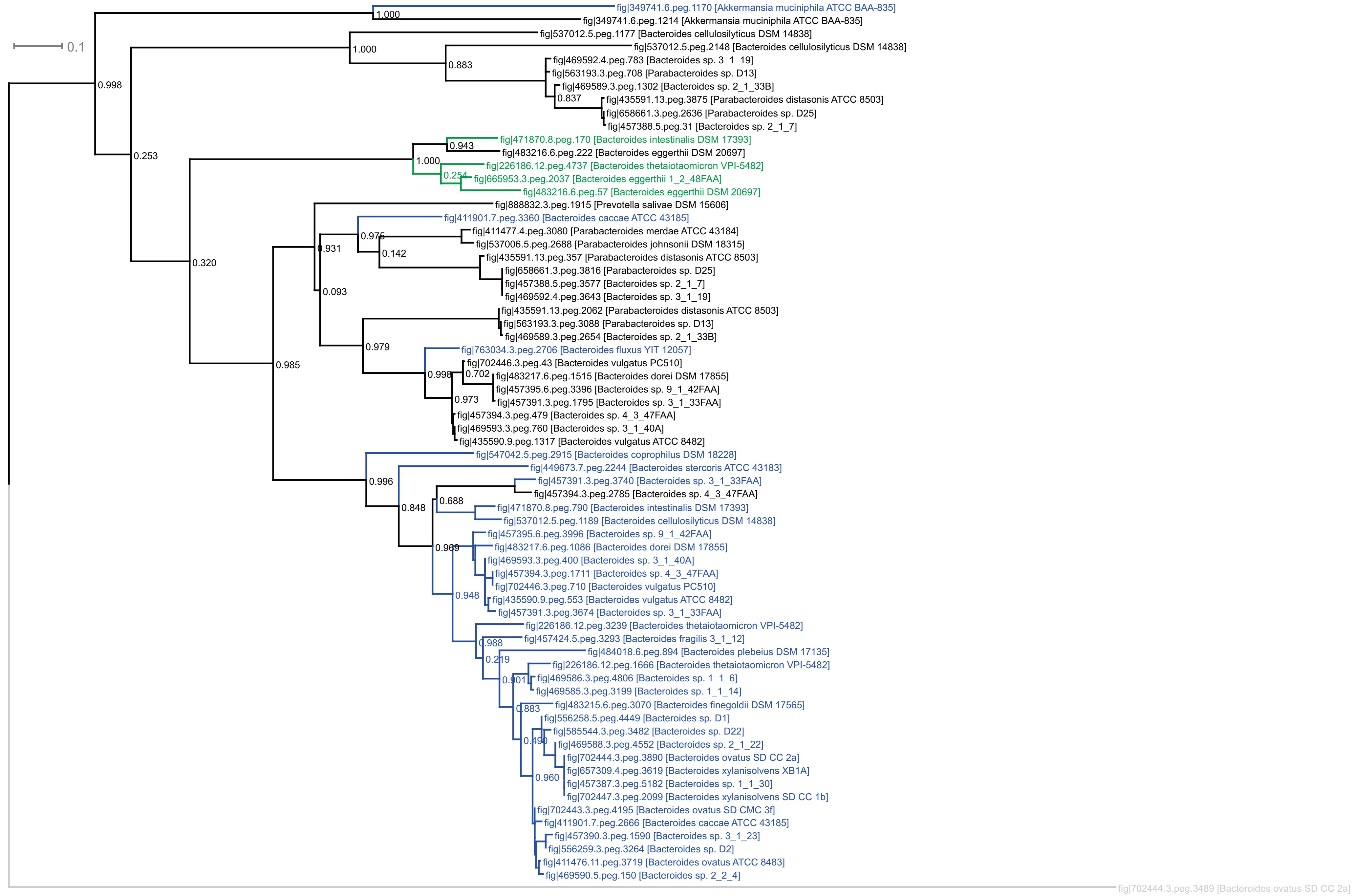

Supplement: Supplementary file 24 [file Image8.PDF]
