# Supplementary material for: Comparative Genomic Analysis of the Human Gut Microbiome Reveals a Broad Distribution of Metabolic Pathways for the Degradation of Host-Synthetized Mucin Glycans and Utilization of Mucin-Derived Monosaccharides
Source: Front Genet. 2017 Aug 29;8:111. doi: 10.3389/fgene.2017.00111 (PMC5583593; doi:10.3389/fgene.2017.00111)

Figure S9. Maximum-likelihood tree for NanE and NanE-II proteins. Functions are shown by colors. The SEED identifiers for proteins are shown; for their sequences, see the file Sequences S1 in the Supplementary materials. Genome names are shown in brackets.

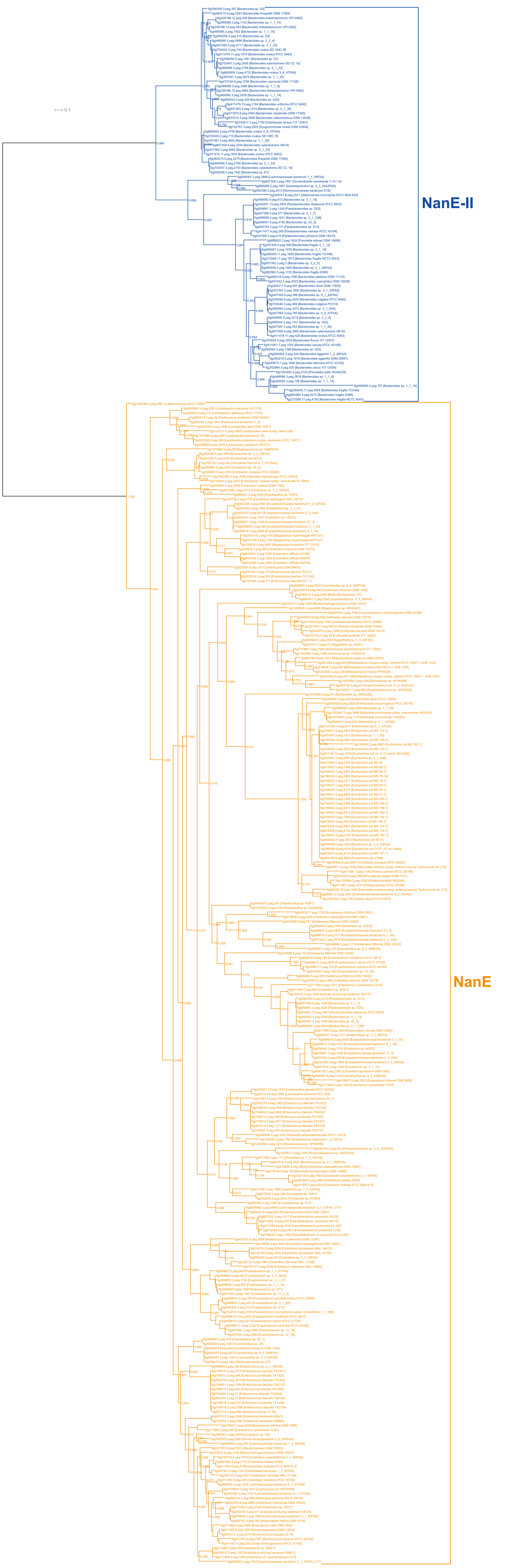

Supplement: Supplementary file 25 [file Image9.pdf]
