# Supplementary material for: Comparative Genomic Analysis of the Human Gut Microbiome Reveals a Broad Distribution of Metabolic Pathways for the Degradation of Host-Synthetized Mucin Glycans and Utilization of Mucin-Derived Monosaccharides
Source: Front Genet. 2017 Aug 29;8:111. doi: 10.3389/fgene.2017.00111 (PMC5583593; doi:10.3389/fgene.2017.00111)

Figure S10. Abundance of mucin glycan-forming monosaccharides in the KEGG database. Number of reactions associated with each monosaccharides is shown in agreement with the KEGG COMPOUND. Number of glycans containing each monosaccharide is shown in agreement with KEGG GLYCAN.

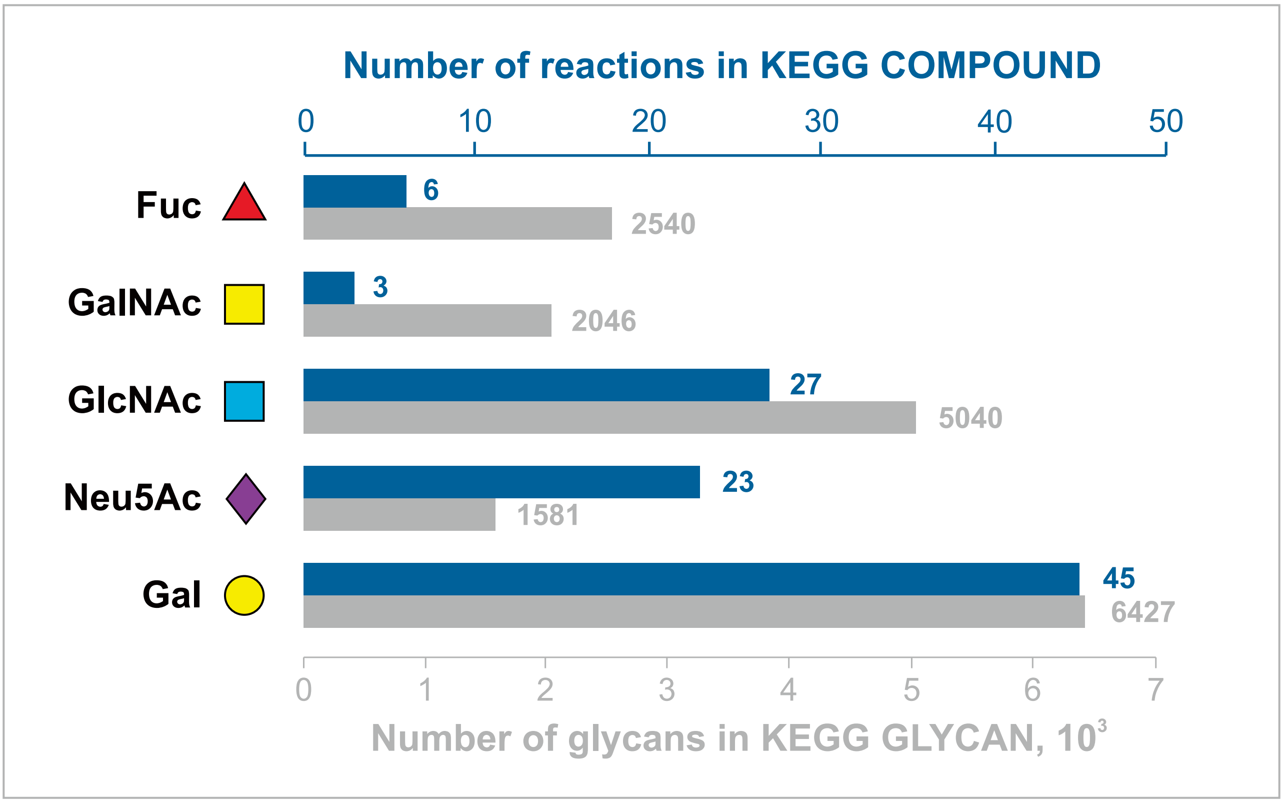

Supplement: Supplementary file 26 [file Image10.PDF]
