# Supplementary material for: Comparative Genomic Analysis of the Human Gut Microbiome Reveals a Broad Distribution of Metabolic Pathways for the Degradation of Host-Synthetized Mucin Glycans and Utilization of Mucin-Derived Monosaccharides
Source: Front Genet. 2017 Aug 29;8:111. doi: 10.3389/fgene.2017.00111 (PMC5583593; doi:10.3389/fgene.2017.00111)

Figure S11. Known structures of mucin glycans detected in human intestine.

1. Structures listed by Podolsky, 1985

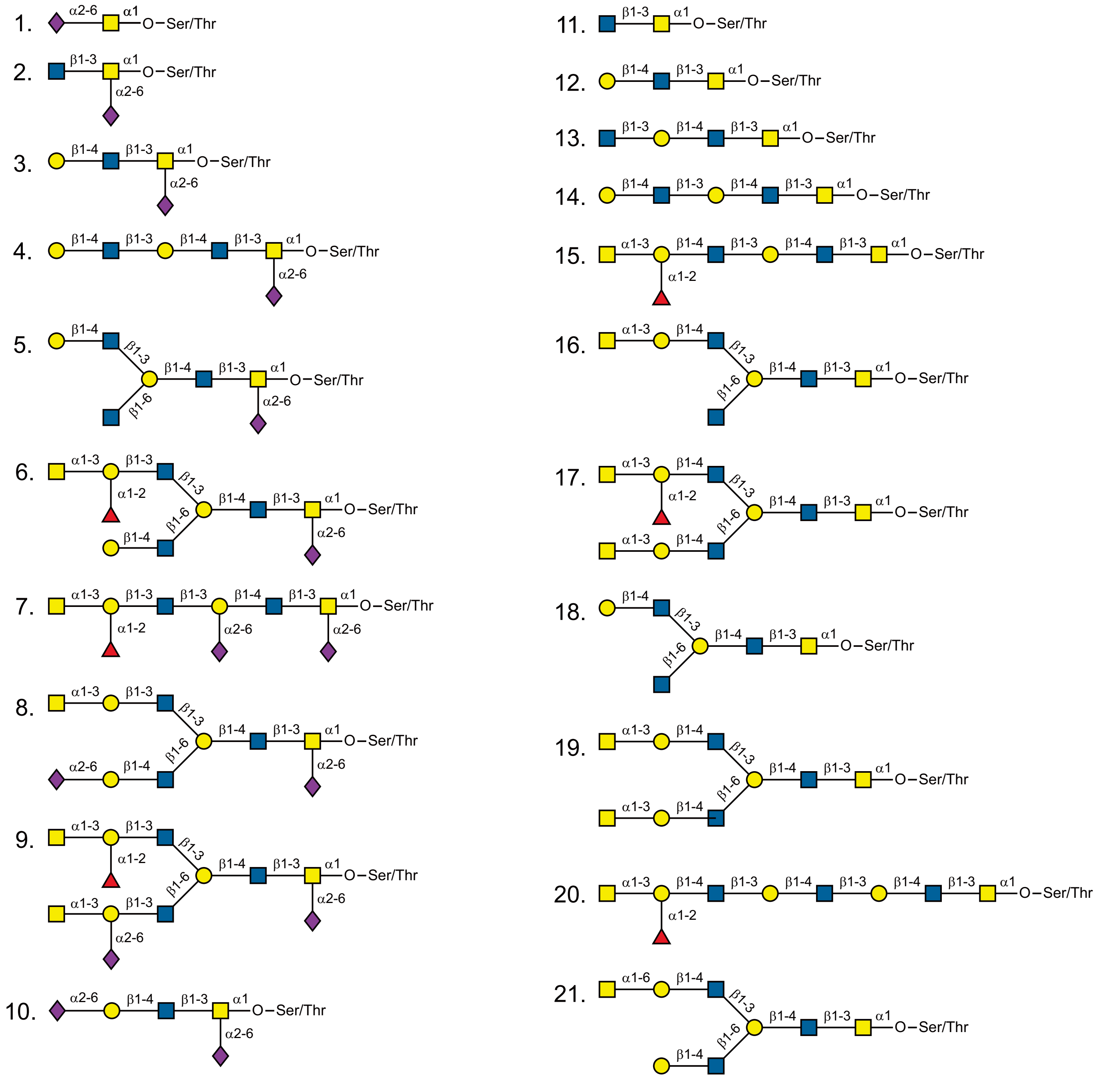

2. Structures listed by Rossez *et al.*, 2012

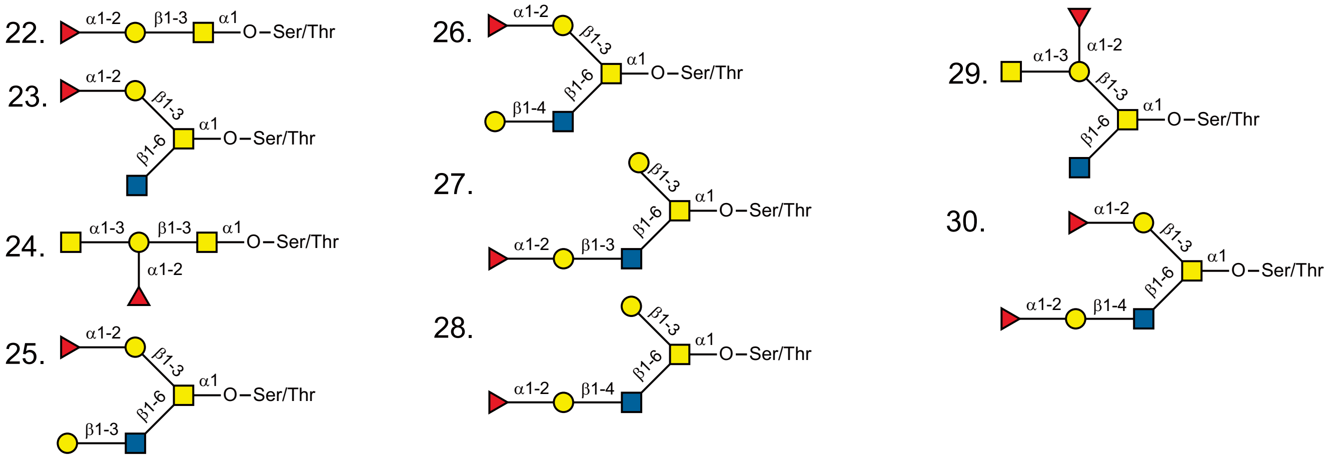

31.

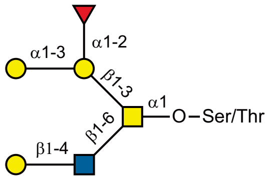

32.

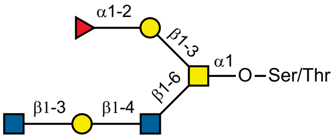

33.

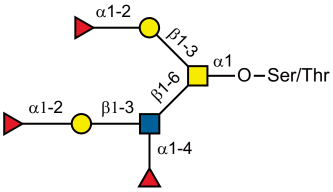

34.

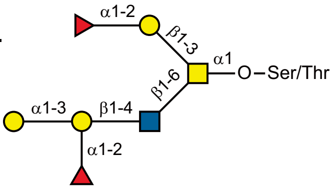

35.

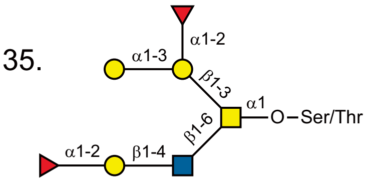

36.

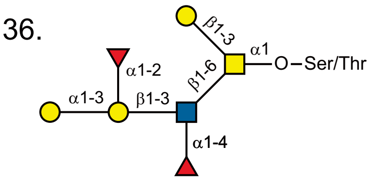

37.

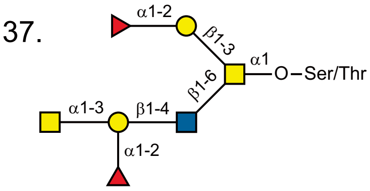

38.

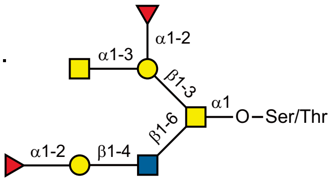

39.

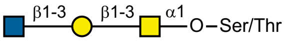

40.

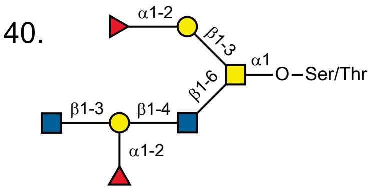

41.

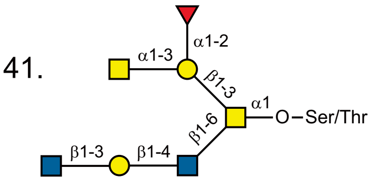

42.

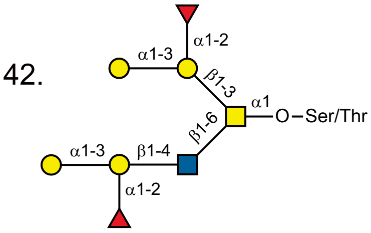

43.

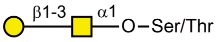

44.

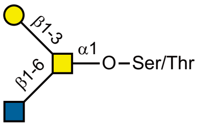

45.

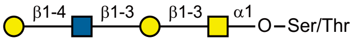

46.

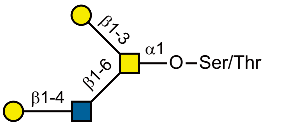

47.

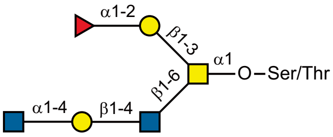

48.

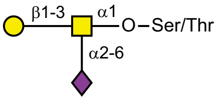

49.

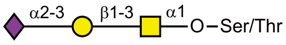

50.

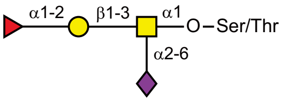

51.

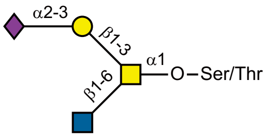

52.

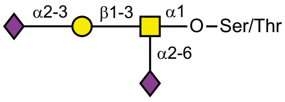

53.

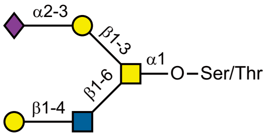

54.

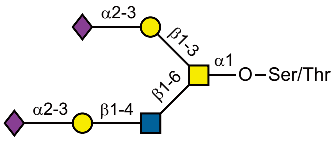

55.

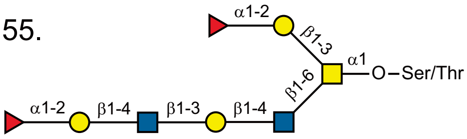

56.

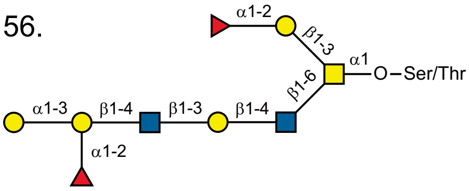

Supplement: Supplementary file 27 [file Image11.PDF]
